# Genomewide Expression and Functional Interactions of Genes under Drought Stress in Maize

**DOI:** 10.1155/2017/2568706

**Published:** 2017-02-23

**Authors:** Nepolean Thirunavukkarasu, Rinku Sharma, Nidhi Singh, Kaliyugam Shiriga, Sweta Mohan, Swati Mittal, Shikha Mittal, Mallana Gowdra Mallikarjuna, Atmakuri Ramakrishna Rao, Prasanta Kumar Dash, Firoz Hossain, Hari Shanker Gupta

**Affiliations:** ^1^Division of Genetics, Indian Agricultural Research Institute, New Delhi 110012, India; ^2^Centre for Agricultural Bioinformatics, Indian Agricultural Statistics Research Institute, Pusa, Library Avenue, New Delhi 110 012, India; ^3^National Research Centre on Plant Biotechnology, Pusa Campus, New Delhi 110012, India; ^4^Borlaug Institute for South Asia (BISA), New Delhi 110012, India

## Abstract

A genomewide transcriptome assay of two subtropical genotypes of maize was used to observe the expression of genes at seedling stage of drought stress. The number of genes expressed differentially was greater in HKI1532 (a drought tolerant genotype) than in PC3 (a drought sensitive genotype), indicating primary differences at the transcriptional level in stress tolerance. The global coexpression networks of the two genotypes differed significantly with respect to the number of modules and the coexpression pattern within the modules. A total of 174 drought-responsive genes were selected from HKI1532, and their coexpression network revealed key correlations between different adaptive pathways, each cluster of the network representing a specific biological function. Transcription factors related to ABA-dependent stomatal closure, signalling, and phosphoprotein cascades work in concert to compensate for reduced photosynthesis. Under stress, water balance was maintained by coexpression of the genes involved in osmotic adjustments and transporter proteins. Metabolism was maintained by the coexpression of genes involved in cell wall modification and protein and lipid metabolism. The interaction of genes involved in crucial biological functions during stress was identified and the results will be useful in targeting important gene interactions to understand drought tolerance in greater detail.

## 1. Introduction

Maize, the third most important food crop in the world after rice and wheat, meets 50–60% of the calorie requirements of people [1]. Considering its importance, increasing maize production under adverse environments has been an active area of research. Drought stress, a major source of environmental stress, lowers crop yields throughout the world [2]. Understanding how plants respond to stress generally is a prerequisite to understanding how they respond to drought at molecular and genomic levels, and a number of promising genes have been identified at the transcriptional level [3].

Abscisic acid (ABA) is the main factor governing stomatal closure, which is affected by regulating guard cell openings [4]. ABA is also an important factor in activating regulatory, enzymatic, and structural genes [5], which play important roles in the perception of stress stimuli, signal transduction, and transcriptional regulatory networks [6]. Transcription factors (TFs) such as MYB,* bZIP*, C2H2, and NAC were expressed to a greater degree in plants under drought [7]. Expression analysis and characterization of TFs have shown being important genes for stress tolerance [8]. TFs follow either an ABA-dependent pathway, which involves the ABA-responsive element binding factors (ABFs) MYC and MYB, or an ABA-independent pathway, which involves drought-responsive element binding (DREB) factors [9]. The role of bZIP in drought tolerance has been studied in many plants [10]. Gene activation and regulation is controlled by kinases and phosphatases such as mitogen-activated protein kinase (MAPK) and calcium kinases. Expression of a number of protein kinases such as histidine kinase, MAPKKK, MAPKK, and MAPK increases in tobacco cells in response to osmotic stress [11]. At the cellular level, maintaining osmotic balance is an important mechanism in combating drought stress [12]. Significant modifications related to toxin removal, water regulation, structural changes, degradation, and repair have been observed in various plants [13]. Several stress-induced genes have been identified and used in engineering drought tolerance in plants [14, 15].

Coexpression network analysis is an important tool in identifying the coexpression of genes in terms of functional association and identifies subsets of genes that are highly correlated with each other in the network [16]. With the availability of large amounts of information from expression analysis, information from multiple experiments can be combined to obtain insights into genes from myriad pathways that have similar expression patterns [17]. Such network-based analysis has been used in studying abiotic sources of stress including drought in rice [18].

The present transcriptome analysis was carried out for identifying differentially expressed genes (DEGs) in drought tolerant and drought sensitive maize genotypes. A number of genes that are expressed differentially in the two genotypes were identified. The coexpression networks showed maximum coexpression of the photosynthetic genes with genes involved in different pathways. Other important modules were discovered, which provide a clearer understanding of the mechanism of drought tolerance associated with the tolerant genotype.

## 2. Materials and Methods

### 2.1. Creation of Stress Treatment

**Figure 1:**
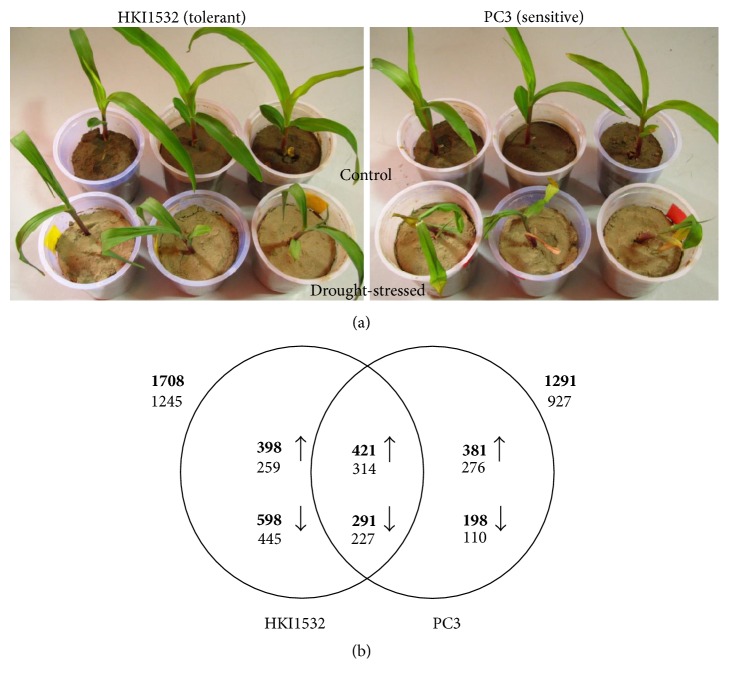
(a) Response of the genotypes to the drought stress. Upper panel-control treatment, lower panel-drought stress treatment. (b) An overview of the differentially expressed genes at *p* ≤ 0.001 with expression levels at least 2-fold those in the control and severe stress stage. Total numbers of genes are shown in bold, below which are the numbers of annotated genes. Genes unique to HKI1532 were greater in number and expressed to a much higher degree compared to those in PC3.

Two inbred lines of subtropical maize (*Zea mays* L.), showing contrasting response to drought tolerance (HKI1532-drought tolerant; PC3-drought sensitive) identified from the previous experiment [19, 20], were sown in plastic cups filled with sandy loam soil in three replications (Figure 1(a)). The first set (stress) was watered daily for 15 days after sowing. Watering was discontinued for the next five days to simulate severe drought stress. The second set (control) was well watered throughout the experiment. Leaf samples for transcriptome assay were collected from both the sets on the 21st day after sowing [21].

### 2.2. Isolation, Labelling, and Hybridization of RNA

Total RNA was extracted from leaf samples (50 mg each) and both quantity and quality of extracted RNA were checked as per Nepolean et al. [19, 20]. For the microarray experiment, Affymetrix GeneChip Maize Genome Arrays (Affymetrix Inc., Santa Clara, California, USA) representing 13,339 genes were used. About 300 ng of total RNA was biotin-labelled and 10 *μ*g of purified fragmented RNA was used for hybridization assay.

### 2.3. Microarray Data Normalisation, Analysis, and Validation

The raw CEL files containing probe intensities from eight chips were generated through GeneChip (GCOS, Affymetrix GeneChip operating software with autoloader, ver. 1.4, manual) and the microarray data was imported into R console using* affy* package [22]. The microarray expression data (accession number: E-MEXP-3992) were submitted to ArrayExpress at the European Bioinformatics Institute. Background correction, normalisation, and probe set summarization were executed using the GeneChip Robust Multiarray Average (GCRMA) algorithm [23]. The differentially expressed genes detection and their significance testing using an empirical Bayes-moderated *t*-test were performed by* limma* package [24]. Probe sets satisfying the criteria of *p* value < 0.001, adjusted *p* value < 0.01, and fold change > 2 as compared to control were considered differentially expressed in response to drought stress. Blast2GO ver. 1.3.3 [25] and PlantTFDB [26] were employed for functional plant-specific annotation of differentially expressed genes. Drought stress specific genes were identified using STIFDB [27] and MapMan tool [28] was used for pathway visualization of stress stage transcripts in the two genotypes. A set of 10 genes from our experiment as well as a published gene (Supplementary Table S1 in Supplementary Material available online at https://doi.org/10.1155/2017/2568706) were taken for qRT-PCR validation of microarray data [19, 20].

### 2.4. Construction of Global and Genotype Specific Coexpression Networks in Response to Drought

Global coexpression networks were created using the raw metadata and* in-house* drought stress microarray data, which comprise 599 samples (527 from NCBI's GEO database, 64 from ArrayExpress Archive of EBI, and 8 from* in-house* drought stress microarray data, platform accession number: GPL4032). All 599 samples were normalised and filtered for outlier samples as per Nepolean et al. [19, 20]. Of the 599 samples, 544 samples passed all the tests and were considered construction of global coexpression network. The* in-house* microarray experimental data were used to generate the drought stress specific coexpression network. The differentially expressed probe sets were mapped to maize loci using maize B73 (ver. 5b.60) gene models (http://www.maizesequence.org/) [19, 20].

Two subsets of data for DEGs from HKI1532 and DEGs from PC3 were generated for the coexpression network analysis. The networks were constructed following the method as described by Ficklin et al. [29]. WGCNA package was used for network construction and module identification. It detected soft threshold (*β*) 5 for both the subsets from HKI1532 and PC3 (Supplementary Figure S1). The eigenvector or eigengene which represents an expression profile for the module and provides a meta-analytic view of the complete network was calculated and clustered using WGCNA (Supplementary Figure S2A). Branches with a merging height of less than 0.2 were merged (Supplementary Figure S2B). Such branches corresponded to modules having eigengenes with a correlation of 0.8 or higher. A set of 174 drought-responsive genes that had actively participated in various molecular and biochemical pathways triggered in response to drought stress in HKI1532 were collected to construct a specific coexpression network. The HKI1542-specific network was generated in the same manner as that used for constructing the global coexpression network.

## 3. Results

### 3.1. Differential Expression Pattern

An 18 k maize genome array was used to profile the expression of transcriptomes in response to drought stress in two genotypes, namely, HKI1532 (5639), which is drought tolerant and PC3 (5146), which is drought sensitive. The number of DEGs in the drought tolerant genotype was significantly greater (*p* ≤ 0.001), and the expression level was 2-fold or higher, than that in the drought sensitive genotype. Under these criteria, 1708 genes were expressed in HKI1532, of which 23% were induced and 35% were repressed. In PC3, of the 1291 genes that were expressed, 29% were upregulated and only 15% were downregulated. Of the 712 genes that were commonly expressed in both the genotypes, more than half (59%) were upregulated. Of the induced DEGs, 48% were unique to HKI1532 (Figure 1(b)).

### 3.2. Gene Annotation

In HKI1532, 73% DEGs were assigned to at least one of the three Gene Ontology (GO) terms, namely, biological process, cellular component, and molecular function; in PC3, corresponding figure was 72%. In the Blast2GO-annotated genes, 46% were upregulated and 54% were downregulated in HKI1532; in PC3, the corresponding numbers were 64% and 36%. Of 463 unannotated genes in HKI1532, 53% were upregulated and 47% were downregulated.

Among the annotated genes, a gene coding for class I heat shock protein (HSP) was the most highly expressed (6542-fold) gene in HKI1532 along with tonoplast intrinsic protein (TIP). In PC3,* endochitinase B* precursor gene, which is a seed chitinase, was the one most highly upregulated (6721-fold). The most downregulated gene in HKI1532 was a transcription factor, namely,* lateral organ boundary domain (LBD)*, along with a gene encoding a transport protein, which was also the most downregulated gene in PC3. Unannotated genes were also among those that had very high transcript levels in HKI1532. These genes included one gene that was expressed more than 2000-fold and three that were expressed more than 200-fold during stress. A few DEGs were collected from GeneChip and were validated in qRT-PCR (Supplementary Figure S3). The results from qRT-PCR showed that though there was a slight variation in fold change of genes the direction of regulation was similar to that of GeneChip results.

**Figure 2:**
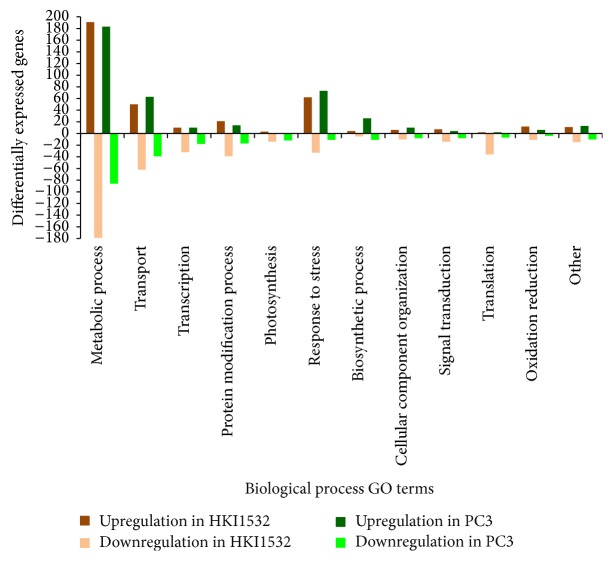
Categorization and regulation of genes in HKI1532 and PC3 according to biological functional categories. Metabolic process, protein modification, signal transduction, and oxidation reduction genes are highly upregulated in HKI1532 as compared to PC3.

**Figure 3:**
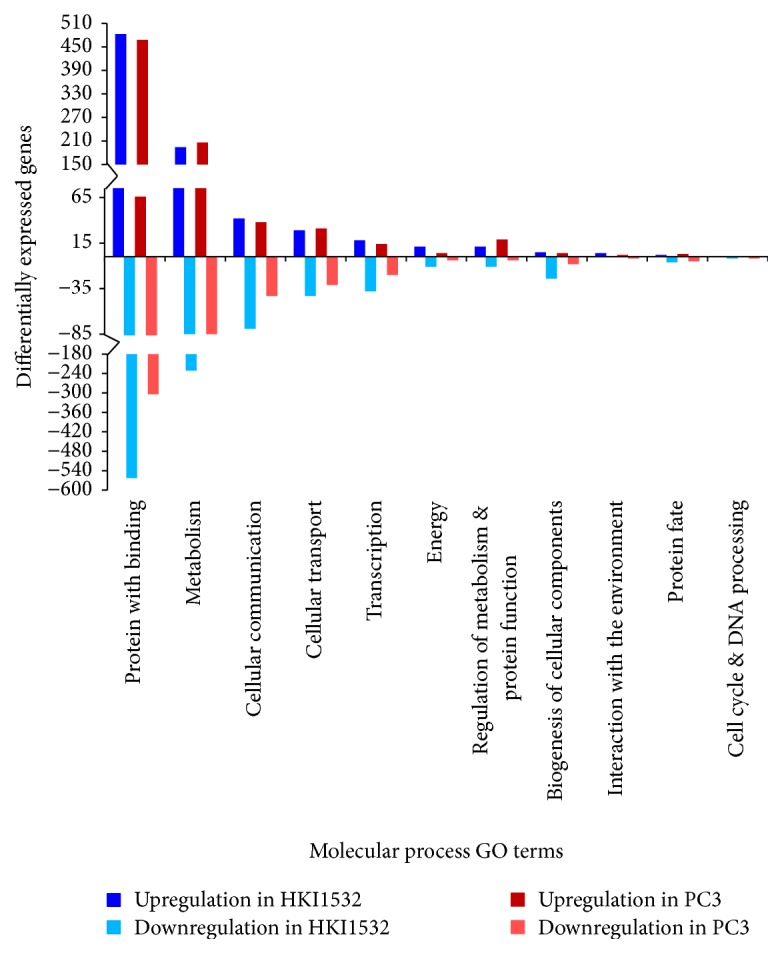
Expression pattern of annotated genes in HKI1532 and PC3 according to MIPS molecular functional categories. Protein with binding and metabolism had the highest number of differentially expressed genes.

According to the biological function GO terms, major genes of both the genotypes were assigned to “metabolic process,” “response to stress,” and “transport” categories (Figure 2). Among these categories, more “metabolic process genes” (50%) were upregulated in HKI1532 than in PC3. Maximum downregulation of genes was also noticed in the same category. Genes involved in “protein modification,” “signal transduction,” and “oxidation reduction” categories were more and more expressed in HKI1532 than in PC3. Categorization of the genes according to MIPS molecular function GO terms placed them into 11 possible molecular categories. For both the genotypes, the maximum number of genes in MIPS classification was placed in “protein with binding” category (Figure 3). Genes involved in “cellular communication,” “transcription,” “biogenesis of cellular components,” and “interaction with the environment” categories were highly expressed in HKI1532 compared with PC3.

### 3.3. Pathway Analysis

**Figure 4:**
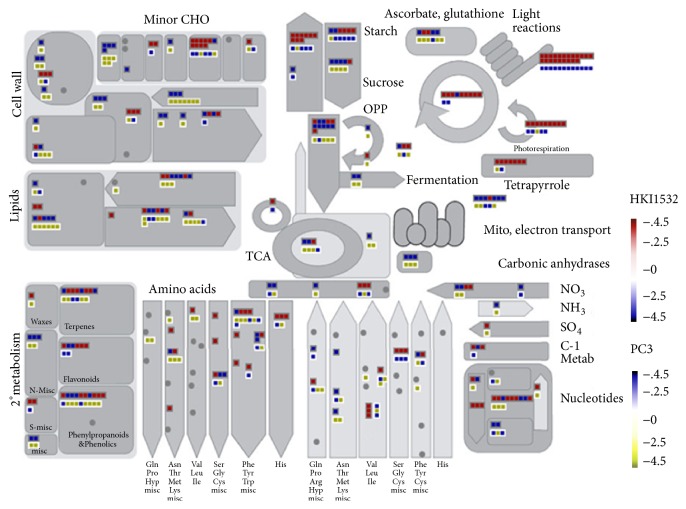
Pictorial representation of genes in HKI1532 and PC3 associated with various MapMan functional metabolism categories under drought stress. Genes are represented as squares having grey and white boundaries. Square with grey boundary represents HKI1532 genes and white boundary represents PC3 genes.

We classified the DEGs expressed in HKI1532 and PC3 into specific metabolic pathways using MapMan tool (Figure 4). For HKI1532, 1501 DEGs were mapped, of which 276 were visible in the metabolism overview map; for PC3, 1115 DEGs were mapped, of which 223 were visible. The “stress” pathway or bin contained the largest number of DEGs (82), followed in that order by “transport,” “signalling,” and “hormone metabolism” in HKI1532. Most of the genes (44%) in the “stress” pathways of HKI1532 were specific to heat stress and six were specific to drought tolerance. Most of the genes involved in hormone metabolism were associated with jasmonate, ethylene, auxin, brassinosteroid, and ABA biosynthesis pathways. The number of such genes was greater in HKI1532 compared to PC3. The two genotypes differed mainly in the number and expression level of the genes involved in photosynthesis and glycolysis. The number of genes associated with photosynthesis in HKI1532 was more than double that in PC3.

### 3.4. Identification of Drought-Specific DEGs and Transcription Factors

Among drought-specific DEGs identified from the stress responsive transcription factor database (STIFDB), 33 showed higher transcript levels in HKI1532. In HKI1532, class I HSP was expressed 18-fold and* TIP* 4-fold, of the corresponding levels in PC3. Twenty-one drought-responsive DEGs mined from the same database were uniquely upregulated in HKI1532 (Supplementary Table S2). Sugar/inositol transporter domain-containing protein-coding gene was expressed 47-fold under stress. Sixty-two genes coded for 24 TFs, of which 44 genes coding for 19 TFs were unique to HKI1532 (Supplementary Table S3) and 14 TF family genes showed higher expression levels in the same genotype.

### 3.5. Global and Specific Coexpression Networks

**Figure 5:**
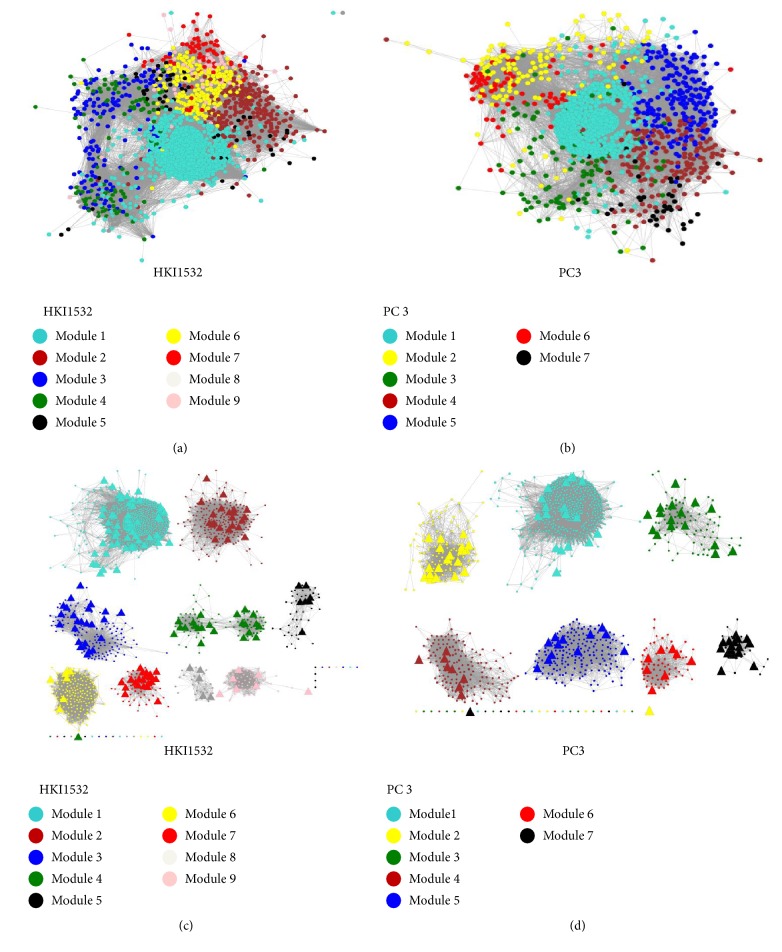
Global (a and b) and module-by-module (c and d) coexpression networks of HKI1532 and PC3 genotypes. Different colours in the global coexpression network (a and b) indicate different modules of the network. Edges (127569 for HKI1532 and 47168 for PC3) indicate significant coexpression of genes in the global coexpression network. Nine and seven modules were formed for HKI1532 (c) and PC3 (d), respectively. Module 1 was the largest module with the highest number of DEGs of both genotypes. Drought-specific DEGs (triangles) were coexpressed with more number of genes in HKI1532.

We created global coexpression networks for both HKI1532 and PC3 using transcriptomic metadata from GEO, EBI ArrayExpress Archive, and in-house microarray expression data (Figures 5(a) and 5(b)). The clustering coefficient for HKI1532 (0.63) was higher for PC3 (0.54). The density of the network was also slightly higher for HKI1532 (0.07) than for PC3 (0.05) but the average path lengths in both the genotypes were comparable (2.4 and 2.6, resp.). Both the networks showed scale-free behaviour, as indicated by the negative linear correlation between the number of edges, or log⁡(*k*), and the probability of finding a node with *k* edges, or *P*(*k*) (Supplementary Figure S4). The number of nodes in HKI1532 and PC3 was 1544 and 1139, respectively, and the edges connecting these nodes were 1 27 569 and 47 168, respectively.

When the coexpression network was studied module by module, nine modules were seen for HKI1532 and seven for PC3 (Figures 5(c) and 5(d)). Module 1 was the largest in both; however, it contained more DEGs in HKI1532 than in PC3 (688 and 455, resp.). Nineteen drought-specific DEGs were part of this module in HKI1532 whereas in PC3 the number was only five. In module 2,* HSP-coding gene* was coexpressed with 19 DEGs but this gene was absent in the coexpression map of PC3. A gene encoding an alpha-amylase isozyme in module 3 was unique to HKI1532 and was coexpressed with 29 DEGs. Four drought-responsive DEGs, namely, 1,4-alpha glucan branching enzyme,* TIP*, cytochrome P450 (*CYP450)*, and an unannotated gene, were included in module 5. These DEGs were coexpressed with 22, 28, 28, and 22 expressed genes and were unique to HKI1532. In module 7, the 70 kDa heat shock cognate protein-coding gene (*hsp70*) was coexpressed with 23 DEGs in HKI1532 but with only 16 in PC3.

**Figure 6:**
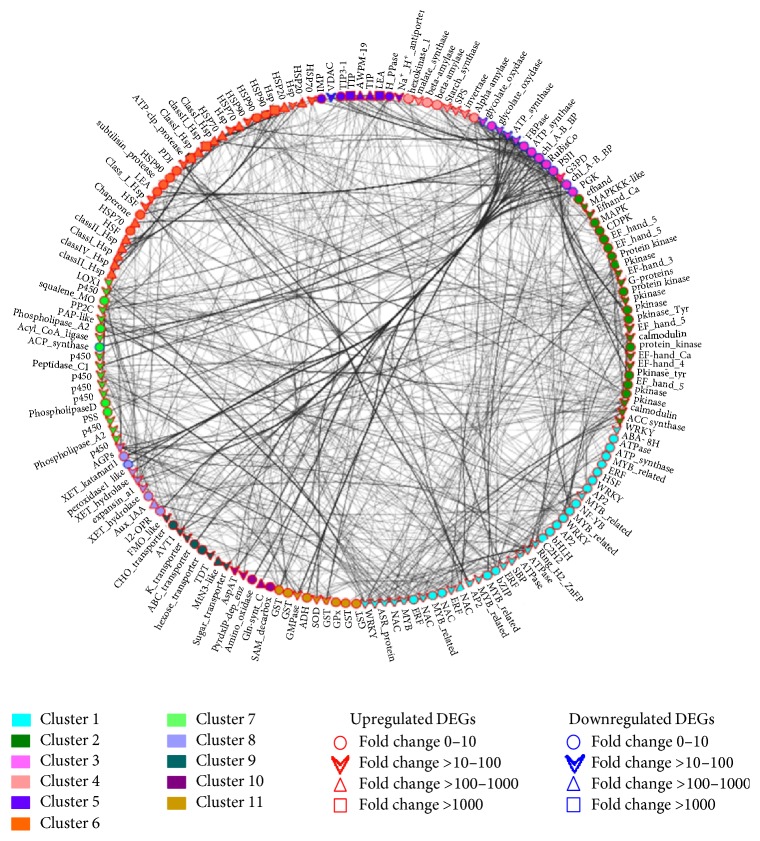
Specific coexpression network of the selected 174 drought-specific DEGs from HKI1532. Every gene fell into one of the eleven clusters, each module representing one specific biological function category. Maximum coexpression in the network was shown by clusters 1, 2, and 3, of which cluster 3 showed the highest degree of coexpression with other genes of the network. Coexpression of genes from different pathways as seen in this network led to drought tolerance in HKI1532.

A more specific coexpression network for 174 selected drought-responsive genes was generated to predict biologically meaningful interactions in HKI1532 (Supplementary Table S4). The network revealed 11 clusters of densely associated genes reflecting coexpression of genes from different pathways (Figure 6). The average path length of the network was 2.73 and the maximum was 4.4. The clustering coefficient of the network fell between 0 and 1. Cluster 4 had the highest degree (23–59) of coexpression with other clusters of the network. A* Myb*-related gene showed the highest neighbourhood connectivity (59), and* ATP synthase* of cluster 3 showed the highest degree of coexpression (59) (Supplementary Table S5).

## 4. Discussion

### 4.1. Global Coexpression Pattern of Genes in HKI1532 and PC3

To understand the genes interactions and their expression patterns under drought stress, we constructed a global coexpression network of all the genes for HKI1532 (Figure 5(a)) and PC3 (Figure 5(b)). The number of coexpressed drought-specific DEGs in HKI1532 was greater than that in PC3, a result that suggests that tolerance of HKI1532 to drought lies in these genes.

The nine modules formed in the global coexpression network of HKI1532 can explain the possible gene-gene interactions. Some unannotated genes were also part of the network. Many drought-specific DEGs were clustered in these modules, with module 1, the largest module of the network, containing the highest number of DEGs. Module 1 showed coexpression of 90 unannotated genes with other functionally annotated genes of HKI1532. Coexpression of the two types of genes, those with unknown functions and those with known functions, probably reveals the involvement of the former in drought tolerance. The majority of the genes in the “stress pathway” from Blast 2GO and MapMan annotations were part of module 7. Thus, coexpression of DEGs with other genes of the network provides a platform for identifying the link between their regulation and expression.

### 4.2. Specific Coexpression Pattern of Drought-Responsive Genes in HKI1532

To reduce the complexity of the global coexpression network for a better understanding of gene interaction, we selected 174 drought-responsive genes from HKI1532 and created a coexpression network for HKI1532 (Figure 6). We found that each of the 11 clusters formed in the coexpression network of HKI1532 represented one major pathway of plant metabolism and regulation. Nearly all the network genes were related to abiotic forms of stress. Comparing this network with the global coexpression network showed that three modules of the global coexpression network accounted for most of the genes: module 1 (57 genes), module 3 (25 genes), and module 4 (23 genes). Module 8 comprised the least number of genes (5). Of the 174 genes selected, 75 genes were uniquely expressed in HKI1532.

Coexpression of signalling genes (cluster 2) and TF (cluster 1) genes with other clusters of the drought-responsive network revealed interlinks in the pathways of these genes and their interdependency. Maximum numbers of genes (35) were part of the first cluster. Out of 35 genes, 27 belonged to TF gene families; most of these TFs were drought specific, well known for their role in stomatal closure* (NAC*,* WRKY*,* ERF, AP2*,* MYB*,* SBP*,* C2H2*, and* NF-YB)*. Plants with more functional* WRKY* are better able to tolerate drought because of ABA-mediated stomatal closure. The higher expression of* WRKY* in HKI1532 (10-fold higher than that in PC3) along with ABA stress ripening protein in the coexpression network confirmed the ABA-mediated regulation of WRKY in stomatal closure.* ERF* and* AP2* transcription factors were expressed in HKI1532 but not in PC3 and were grouped with* NAC*,* MYB*,* SBP*,* bZIP*, and* bHLH *(all with much higher expression levels) in the first cluster. This kind of grouping provides ample evidence of the involvement of these genes in morphological and molecular changes in HKI1532 under drought [30].

Most of the signalling and phosphoprotein cascade genes were part of the second cluster. Major members of this cluster, the third largest in the network, were proteins of the EF-hand family, members of the auxin-responsive IAA family, and calcium-dependent protein kinases. The expression levels of all of them were much greater in HKI1532, and their roles in combating drought stress are well documented [11]. Coexpression of this cluster with photosynthetic genes showed their mutuality in terms of their expression.

Our study found brassinosteroid receptors (BRs) and related genes were downregulated in both HKI1532 and PC3. These are new family of plant hormones having crosstalk with other growth promoting phytohormones at different plant growth and developmental stages. BRs at molecular levels are involved in cellular expansion, growth, and development of individual cells through anabolic processes that ensure plant development and efficient flowering to complete life cycle. With onset of hydropenia plants quarantine all the anabolic process with commensurate reduction of BRs gene function and shift to early flowering by invoking flowering locus-C (FLC) function. Additionally, BRs alleviate oxidative damage by expressing ROS scavenging genes in water stress [31] and stress tolerating ability of BR lies in its crosstalk with other phytohormones such as ABA hormone [32]. In our study, ABA (a growth retarding hormone) synthesizing and signalling molecules are upregulated in stress to attenuate BRs gene expression.

Although photosynthetic genes of the third cluster were mostly downregulated in PC3, they were downregulated in HKI1532 to much lesser extent. Clustering of photosynthetic genes with the signalling genes and TFs showed their important and mutually reinforcing role in drought tolerance. It can be assumed that downregulation of photosynthetic genes is directly connected with inducing genes in other regulatory pathways involved in different functions. The interaction of genes involved in osmoregulation, stress responsive transcription factors, and other metabolic pathways mentioned in the following discussion revealed that HKI1532 was better adapted to water stress at a lower rate of photosynthesis in order to maintain the crucial biological functions.

Plants store carbohydrates as reserves, to be used when energy supply is limited. Major genes of cluster 4 were coexpressed with active genes for starch degradation* (amylases*,* hexokinase*, and* invertase)* and sucrose synthesis (*SPS *and* starch synthase*), which indicates strong regulation of carbohydrate metabolism in HKI1532:* SPS *and* starch synthase* were suppressed in PC3 but expressed 7-fold to 8-fold higher in HKI1532. Osmoregulation and ion homeostasis governing water intake in plants are important mechanisms for coping with drought.* LEA*,* TIP*, and* AWPM *family proteins were important members of the fifth cluster involved in osmoregulation.* LEA* family proteins in HKI1532 were expressed 1300-fold and* TIP* was expressed 4800-fold (but only 1180-fold in PC3). Many studies have reported such simultaneous increase in the expression of these genes with increase in ABA levels [33]. Their coexpression pattern indicates that their regulation is influenced by clusters 1, 2, and 3.

It is well known that HSPs are accumulated as a response to stress, whether from abiotic sources or from biotic sources, and act as a defence system [34]. The sixth cluster was the second largest cluster, containing members of HSP families, all of which are drought specific and belonged to the “response to stress” GO category. Along with HSPs, members of protease family and chaperones such as protein disulphide isomerase* (PDI)* were also included in this cluster. Their coexpression with other genes indicates that their regulation and the genes under their control are highly complex but important to a plant's responses to stress.

The seventh cluster comprising 18 genes was highly connected with clusters 1, 2, and 3. The connections are visible in the form of the dense edges connecting these genes to each other (Figure 6). Phosphatidic acid phosphatase* (PAP)* and* P450* showed the highest coexpression.* PAP* catalyses dephosphorylation of PA, generating DAG and Pi, which regulate the downstream lipid signalling pathways.* PAP*,* PLA2*, and* p450* were highly expressed in HKI1532 (more than 10-fold) but much less in PC3. It was evident from the coexpression network that* PAP*,* P450*, and* LOX* were coexpressed with ABA and stomatal closing TFs* (WRKY, NAC, MYB, C2H2, AP2*, and* ERF)*. Thus, genes in cluster 7 showed interconnections between their regulation and expression and were assigned to the broad category of lipid biosynthesis and hydrolysis.

Additionally, squalene monooxygenase is exclusively upregulated (8 folds) only in HKI1532 (tolerant genotype). It is an important enzyme involved in sterol biosynthesis in plants, oxidizing squalene to 2,3-oxidosqualene, an intermediate for cell membrane triterpenoids for steroids synthesis. Sterols are isoprenoid-derived lipids that play vital roles in plant growth and development and seem to have an important role in plants to regulate many of its metabolisms in stress condition [35]. Sterol significance is proved by a study in which dry2/sqe1-5 mutant having altered root sterol composition was hypersensitive to drought and had altered stomatal response and ROS production. HKI1532 expressing sterol synthesizing gene indicates a development of tolerance mechanism in plants by expressing their steroid synthesizing gene. Moreover, sterol may influence the ROS production which can be considered as ROS scavenging genes that were also upregulated in the HKI1532 than PC3.

Flavonoid acts as an antioxidant in plants under stress conditions. Modulation of flavonoid pathway and increased flavonoid content in response to drought stress is well known [36] and our study also reveals that flavonoid synthesis is another water stress tolerating mechanism as flavonoid monooxygenase involved in flavonoid biosynthesis was exclusively upregulated only in HKI1532. Besides photosynthesis and toxin elimination, it has been recently reported that plants quarantine all the anabolic component of metabolic activities during hydropenia [37]. Since monooxygenases (Moxs) are involved in fatty acid biosynthesis corresponding to counter the drought-induced damage of the cell membrane, the cassette of Moxs is prominently upregulated and our result is commensurate with the previous finding.

The coexpression of cluster 7 with cell wall components and cell-wall-modifying genes of cluster 8 reveals their correlation since membrane phospholipids are important to maintaining the integrity of cell walls. The* 12-OPR* is involved in jasmonate signalling, an important gene expressed under stress [38], accumulated as the levels of ABA increase. The level of expression of* 12-OPR* in HKI1532 (8.1-fold) was nearly double that in PC3 (4.3-fold). Flavonoid monooxygenase (*FMO*) expressed only in HKI1532 induces cell expansion. Cluster 8 was highly coexpressed with cluster 7 and cluster 4, which highlights their interconnected functions in drought tolerance.

Drought tolerant plants maintain turgor pressure at low water potentials by increasing the number of solute molecules in the cell. Potassium being the most abundant cation in plants is involved chiefly in osmoticum mediated cell expansion, membrane permeability, and drought resistance. Additionally, hydropenia elicits considerable disturbance to K^+^ homeostasis and provokes expression of K^+^ channels and transporters to maintain K^+^ homeostasis [39]. All transporter families of genes comprising nine genes are presented in the ninth cluster in our study. Among them,* MtN3* and sugar transporter membrane proteins of this cluster were among the most highly expressed genes in the network and were also expressed to a much greater degree in HKI1532 than in PC3. Higher expression of these transporter genes and their coexpression with clusters 1 and 3 underscored the importance of various transporter genes in tolerating drought stress. K^+^ transporter gene in PC3 was upregulated 6.3-fold while the upregulation was 11.4-fold in HKI1532. Upregulation of these genes in both the genotypes but with high fold change in the tolerant one indicates the importance of ion transport across membrane and thus participation in maintaining osmoregulation in the stress plant.

Aldehyde dehydrogenase* (ADH)* was highly expressed in HKI1532 and not expressed at all in PC3, a finding consistent with that of Gao and Han [40] who studied rice under drought conditions. The most important genes, namely, those for ROS scavenging and the superoxide dismutase (*SOD*) activity, were also expressed to a much higher degree in the present study. Placing of* ADH* and* SOD* in the same cluster with other detoxifying and ROS scavenging genes emphasizes the activation of the elimination mechanism in plants to reduce the toxic effect of ROS and of toxin accumulation. ROS generated by various metabolic pathways such as photosynthesis, photorespiration, and lipid peroxidation are kept under control by catalase and peroxidases [41].

Cluster 9, a small cluster comprising genes of amino acid regulation, was part of the last cluster of the network. Cluster 9 showed the highest coexpression with cluster 3 and cluster 5.* Glutamine synthetase* in this cluster was among the most expressed genes in HKI1532. Cys/met PLP dependent enzyme is involved in synthesizing methionine and cysteine, which are nutritionally important amino acids for plants. Also, methionine is an important substrate for ethylene synthesis [42].

### 4.3. Drought-Responsive Transcriptome Regulation and Expression

Based on the specific coexpression network of drought-responsive genes in HKI1532, we advance a hypothesis to explain the interaction and regulation of these genes under drought. A complex web of signalling is triggered in drought stress, which relays messages through the plasma membrane to the cell, activating a signalling cascade that enables a plant to regulate its growth and metabolism accordingly [11].

#### 4.3.1. Effect of Drought on Photosynthesis and Other Metabolic Pathways

**Figure 7:**
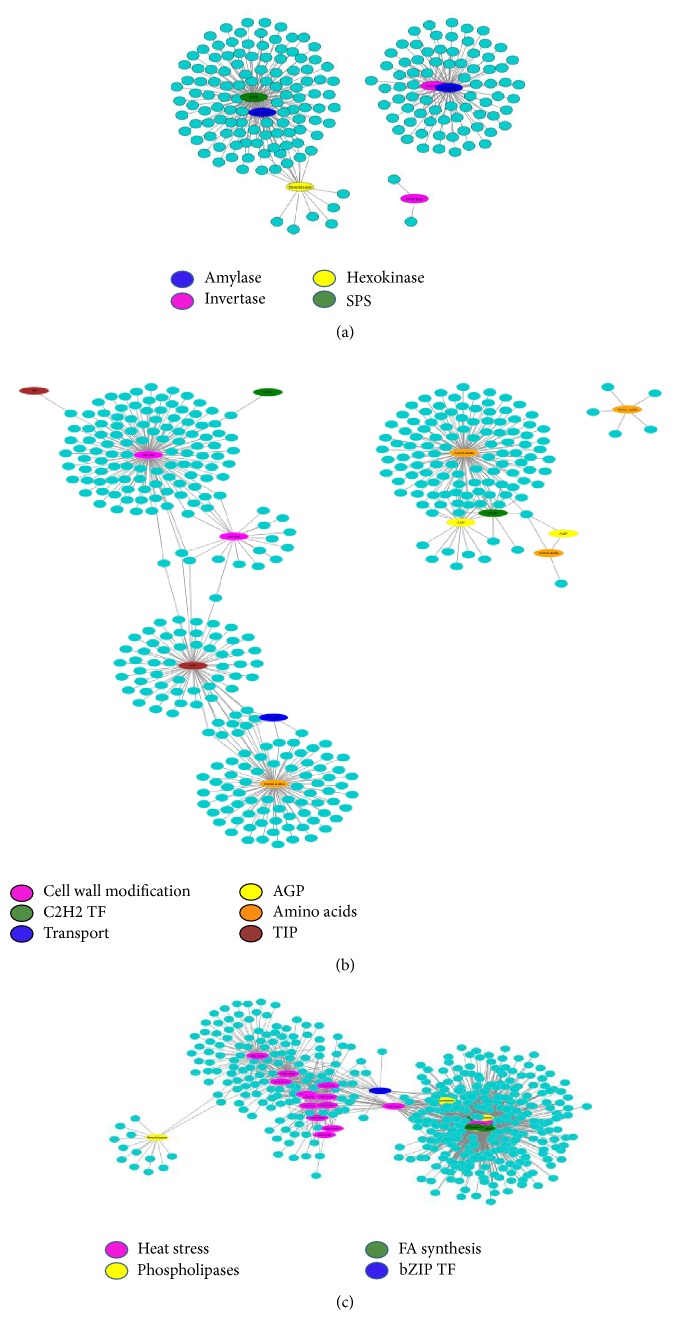
Coexpression network of candidate genes in different pathways. (a) Coexpression of genes involved in photosynthesis and other metabolic pathways, (b) in water balance, and (c) in combating dehydration.

*Phosphoglycerate kinase*,* glycolate oxidase*,* ATP synthase*,* RuBisCo small subunit*, and* chlorophyll A-B binding *proteins were the important photosynthetic genes inhibited in both HKI1532 and PC3. This validates the assumption that although stomatal closure reduces water loss it also lowers photosynthetic efficiency because the availability of CO_2_ is also reduced [43]. The reduced photosynthetic rate limits the carbon skeleton and nutrients accessible for plant metabolism. This deprivation compels cells to modify other metabolic pathways accordingly to meet the energy demand. This process is also observed in HKI1532. Degradation of starch, lipids, and proteins (discussed later) is the main response observed in this connection. Starch, the major carbohydrate reserve of plants during germination, is catabolized to glucose, maltose, and other oligosaccharides by* amylase *[44]. The coexpression of* amylase*,* invertase*,* hexokinase*,* SPS*, and sugar transporter genes of clusters 1, 3, 4, and 9 (Figure 7(a)) ensures the availability of low-molecular-weight carbohydrates [45]. Increase in the expression of these gene transcripts under drought stress was also reported in other studies [3]. Genes involved in starch synthesis were downregulated and sucrose synthesizing genes were highly upregulated in HKI1532 in terms of both number and the degree of expression.

#### 4.3.2. Strategy for Maintaining Water Balance in Drought Stress

Maintaining low water potential is a rescue process in plants under stress from drought or salinity [46]. A member of aquaporin family, namely,* TIP*, regulates the movement of water and of small solutes by increasing the permeability of vacuolar membranes. This increased permeability helps in osmotic buffering, allowing plants to maintain a low water potential [47]. Osmolytes such as sucrose, amino acids, and polyamine also lower the plant water potential to match the soil water potential, facilitating water uptake. A group of genes responsible for osmolyte synthesis and* TIP* were found to be highly upregulated in HKI1532. LEA proteins, which form an evolutionarily conserved group of hydrophilins, are regulated by abscisic acid and C2H2 TF in maintaining osmotic balance. These proteins also stabilize the cells under drought stress [48]. The high coexpression metabolic genes highlight the mechanism that promotes water use efficiency in HKI1532 (Figure 7(b)). In addition to maintaining a low water potential, differential regulation of growth has also been considered as an adaptation strategy of plants because slowing down growth in the distal elongation zone of roots ensures uninterrupted water uptake from soil [49]. Coexpression of the cell wall protein* (AGP*s) and the cell wall expansion gene* (expansin and XET)* supports induced growth in roots. Cell wall stiffening genes* (XTH)* were grouped along with the abovementioned genes in the same cluster, a grouping that supports the hypothesis that growth is differentially regulated in apical and basal regions of the root elongation zone [50], making HKI1532 better adapted than PC3 to drought stress.

#### 4.3.3. Protective Mechanisms Adapted for Combating Dehydration

Lipids and fatty acids are the main components of cell wall membranes and their interaction facilitates membrane stability. Dehydration degrades cell membranes by reducing the lipid and fatty acid (FA) content of cell membranes [51]. Increased expression of lipolytic genes along with that of lipid and FA synthesizing genes highlights the modified lipid metabolism and degradation in HKI1532 (Figure 7(c)). This increased expression in turn affects the structural integrity of cells. Although the lipolytic activity of PLA2, D, and LOX results in the loss of membrane function, the molecules produced during lipid degradation act as signalling molecules and initiate stress-related responses, a sequence of events also noticed in HKI1532 in the form of the coexpression of signalling genes and other pathways (Figure 6) [51]. Lipid and FA synthesizing genes such as* ACP synthase*, phosphatidylserine synthase* (PSS)*, and* PAP* were differentially expressed in the present study, showing that despite degradation, HKI1532 is able to maintain the synthesis of lipids and FA for protecting the cell membrane by stabilizing its lipid contents. Water deficit also upsets the processing and folding of proteins, which ultimately results in the accumulation of large amounts of nonfunctional, damaged, and wrongly folded proteins [34]. Consequently,* chaperones*,* HSPs*, and proteases were induced in HKI1532 to a very high degree (more than 4000-fold), indicating the heightened response of HKI1532 to protein folding. TFs such as* bZIP* and* NF-Y* and kinases such as* MAPK* and* MAPKKK*, coexpressed significantly with HSPs in HKI1532 (Figure 7(c)), are involved in the degradation of unfolded proteins. At the same time, the greater expression of protease genes promotes the mechanism to eliminate nonfunctional proteins in HKI1532. The intermediates of some of these pathways such as carbohydrate metabolism, lipid metabolism, and photosynthesis lead to increased production of ROS and toxic substances [52]. ROS are beneficial in small amounts but harmful to plant growth and survival when accumulated in large quantities. In response, glutathione s-transferase* (GST)*,* SOD*, and* ADH* were upregulated in HKI1532 to control the levels of toxins and ROS.

## 5. Conclusions

The genomewide transcriptome assay revealed the differential expression of genes during water stress in HKI1532, a drought tolerant genotype, and PC3, a drought sensitive genotype. The global coexpression network identified cooperation among genes in dealing with drought stress. The specific coexpression network of drought-responsive genes of HKI1532 explained the relation between genes of multiple pathways including photosynthesis, osmotic adjustments, and metabolism. The genes identified from this experiment will be useful for selecting candidates for breeding drought tolerance in maize. The selected genes can be validated in segregating mapping populations as well as in marker-assisted backcross breeding approach.

## Supplementary Material

Supplementary Figure S1: Analysis of network topology for various soft-thresholding powers.
Supplementary Figure S2: Clustering of module eigenvectors (ME) of the co-expression networks comprising nine and eight modules for HKI1532 and PC3 respectively.
Figure S3: Validation of selected DEGs from GeneChip by qRT-PCR tested in HKI1532 (Tolerant) and PC3 (Sensitive) genotypes. ZmNAC18, a previously published drought-responsive gene, is used as an external control. Genechip values are given on the label (orange).
Supplementary Figure S4: Scatter plot between the number of edges (log(k)) and the probability of a node having k edges (P(k)).
Supplementary Table S1: Selected DEGs from GeneChip for validation by qRT-PCR.
Supplementary Table S2: Drought-responsive DEGs exclusively up-regulated in HKI1532.
Supplementary Table S3: Transcription factors unique to HKI1532 and their expression values under water stress.
Supplementary Table S4: A set of 174 selected drought-responsive genes from HKI1532 and their co-expression network groups.
Supplementary Table S5: Statistical parameters of the co-expression network of genes specific to drought tolerance.
