# Supplementary material for: Genomewide Expression and Functional Interactions of Genes under Drought Stress in Maize
Source: Int J Genomics. 2017 Feb 23;2017:2568706. doi: 10.1155/2017/2568706 (PMC5343257; doi:10.1155/2017/2568706)
Supplement: Supplementary file 1 — Supplementary Figure S1: Analysis of network topology for various soft-thresholding powers. Supplementary Figure S2: Clustering of module eigenvectors (ME) of the co-expression networks comprising nine and eight modules for HKI1532 and PC3 respectively. Figure S3: Validation of selected DEGs from GeneChip by qRT-PCR tested in HKI1532 (Tolerant) and PC3 (Sensitive) genotypes. ZmNAC18, a previously published drought-responsive gene, is used as an external control. Genechip values are given on the label (orange). Supplementary Figure S4: Scatter plot between the number of edges (log(k)) and the probability of a node having k edges (P(k)). Supplementary Table S1: Selected DEGs from GeneChip for validation by qRT-PCR. Supplementary Table S2: Drought-responsive DEGs exclusively up-regulated in HKI1532. Supplementary Table S3: Transcription factors unique to HKI1532 and their expression values under water stress. Supplementary Table S4: A set of 174 selected drought-responsive genes from HKI1532 and their co-expression network groups. Supplementary Table S5: Statistical parameters of the co-expression network of genes specific to drought tolerance. [file 2568706.f1.pdf]

Functional interactions of drought-responsive genes indentified through genome-wide expression assay in maize

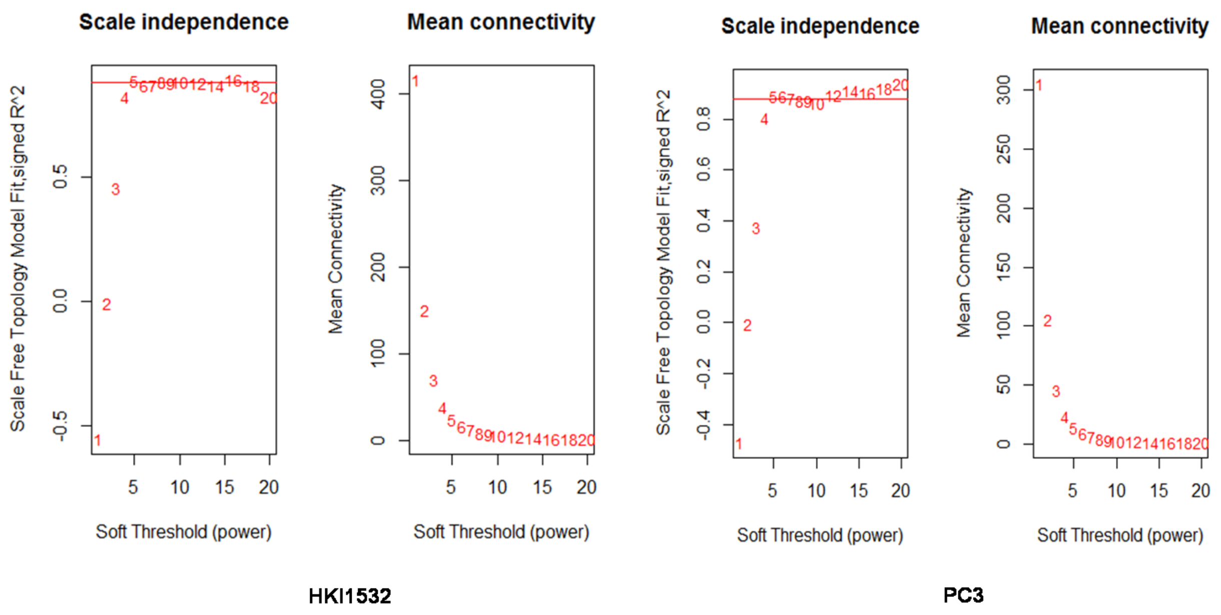

Supplementary Figure. S1. Analysis of network topology for various soft-thresholding powers.

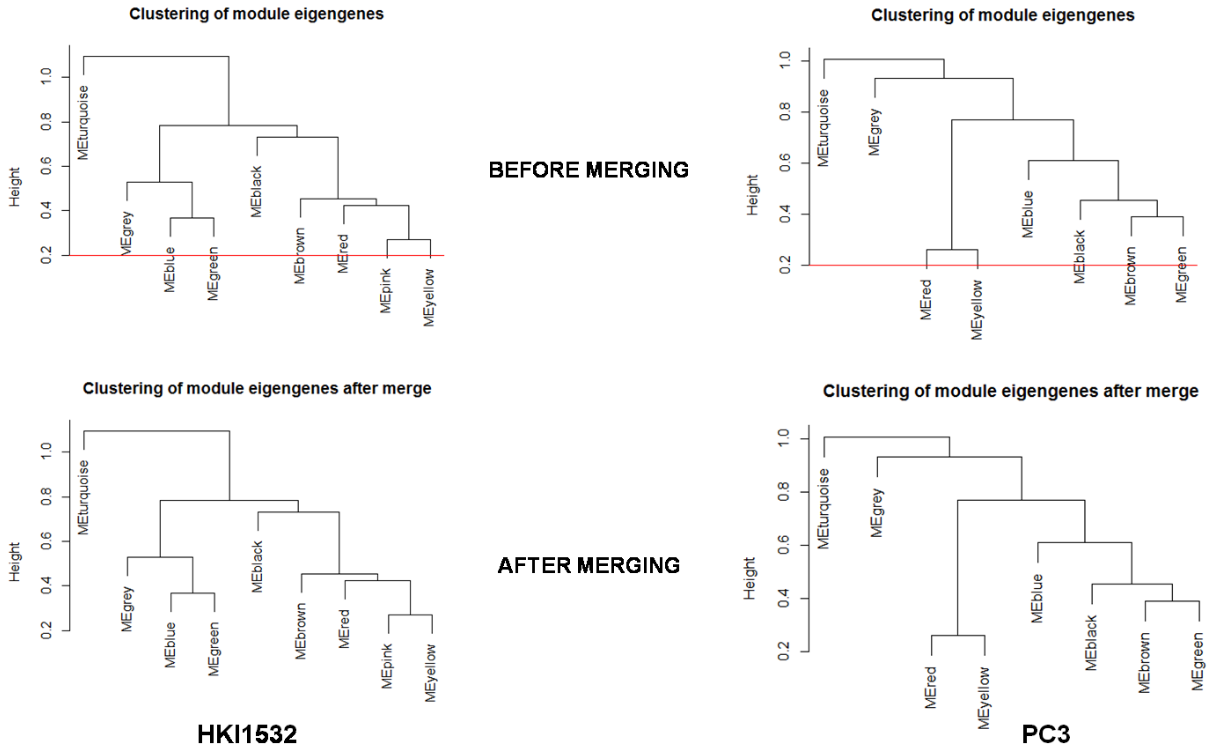

Supplementary Figure. S2. Clustering of module eigenvectors (ME) of the co-expression networks comprising nine and eight modules for HKI1532 and PC3 respectively.

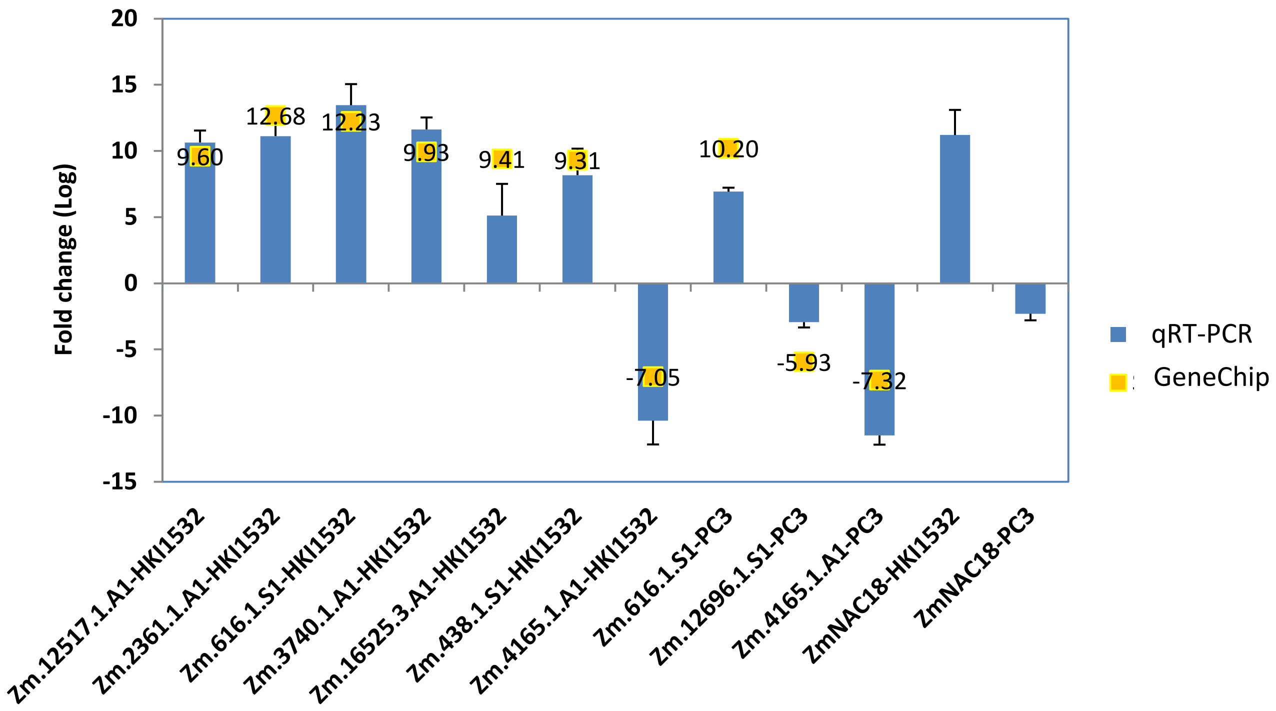

Figure S3. Validation of selected DEGs from GeneChip by qRT-PCR tested in HKI1532 (Tolerant) and PC3 (Sensitive) genotypes. ZmNAC18, a previously published drought-responsive gene, is used as an external control. Genechip values are given on the label (orange).

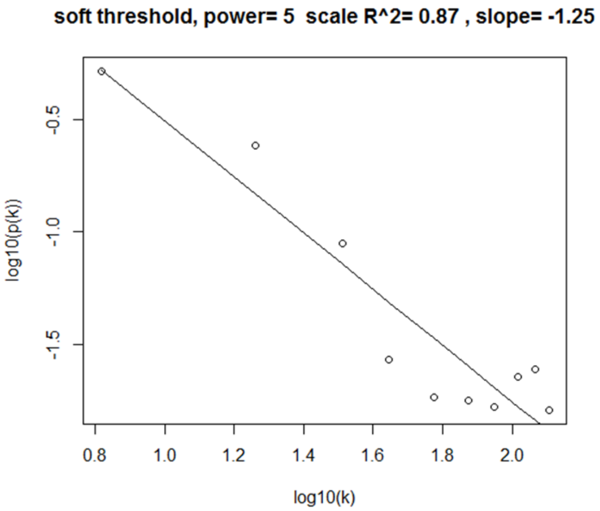

HKI1532

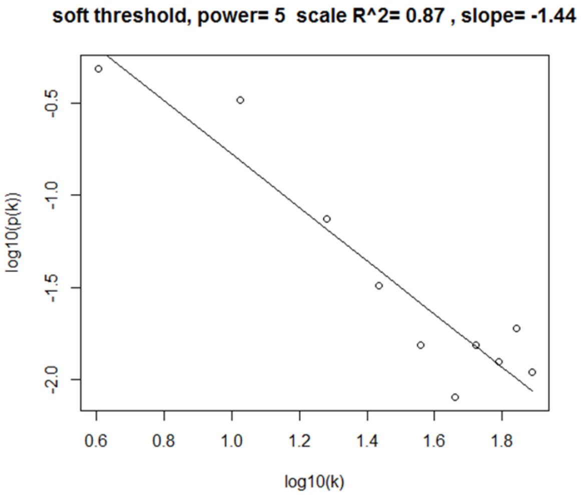

PC3

Supplementary Figure S4. Scatter plot between the number of edges (log(k)) and the probability of a node having k edges (P(k)).

Supplementary Table S1. Selected DEGs from GeneChip for validation by qRT-PCR.

Probe set ID	Genotype studied	Annotation	Primer sequence	Primer T_m
Zm.12517.1.A1_at	HKI1532	kda class iii heat shock protein	F: AGGTCACCATCGCCTAGAA	62.4
			R: AAAGAGCACTCCACCCATTC	62.2
Zm.2361.1.A1_at	HKI1532	heat shock protein	F: AAGAACGTCCAGATCACCGGCTA	60.6
			R: ATCACTGCACACAACGGGAGTACA	60.2
Zm.616.1.S1_at	HKI1532	tonoplast intrinsic protein	F: CGCCAGAGGACTACTAGCTTGAAA	57.9
			R: ACCTGATTCACCGTCTCCAACACA	61.3
Zm.3740.1.A1_at	HKI1532	kda class iv heat shock protein precursor	F: CGTCCAAGGCTGAGATGTGAATGT	61.0
			R: AACGGGATCAATACGCACCGGATA	63.9
Zm.16525.3.A1_x_at	HKI1532	heat shock protein 82	F: GTATGATCAGGTCCGCCATAG	61.6
			R: GTCAGAGACCACAACCTTCTC	61.8
Zm.438.1.S1_at	HKI1532	heat shock protein 101	F: TGTCTCTCTCTTACGATCCGGTGT	57.8
			R: ATCTCCTCCTGGATCAGCATCTTC	58.6
Zm.4165.1.A1_at	HKI1532	phosphoenolpyruvate carboxykinase	F: AAGAAGAACTTCGAGGTGTTCGCC	60.8
			R: CGTCGTCGTCGTAATCATCTTCAC	59.4
Zm.616.1.S1_at	PC3	tonoplast intrinsic protein	F: CGCCAGAGGACTACTAGCTTGAAA	57.9
			R: ACCTGATTCACCGTCTCCAACACA	61.3
Zm.12696.1.S1_at	PC3	myb family transcription expressed	F: TGCTCGATTCTTCGTCATGGAAC	61.2
			R: CAGCGTCACAATCAATGCGG	60.5
Zm.4165.1.A1_at	PC3	phosphoenolpyruvate carboxykinase	F: AAGAAGAACTTCGAGGTGTTCGCC	60.8
			R: CGTCGTCGTCGTAATCATCTTCAC	59.4
ZmNAC18* (Control)	HKI1532	Drought-responsive transcription factor	F: GAAGAACGAGTGGGAGAAGATG	62.1
			R: ACGAGTGCGAGTGTGATTG	62.3
ZmNAC18* (Control)	PC3	Drought-responsive transcription factor	F: GAAGAACGAGTGGGAGAAGATG	62.1
			R: ACGAGTGCGAGTGTGATTG	62.3

*Kaliyugam S, Rinku S, Krishan K, Yadav SK, Firoz H, Nepolean T (2014). Genome-wide identification and expression pattern of drought-responsive members of the NAC family in maize. Meta Gene 2:407-417.

Supplementary Table S2. Drought-responsive DEGs exclusively up-regulated in HKI1532.

Probe ID	Sequence description	log FC	Absolute FC	*p* value
Zm.622.1.S1_at	Tonolplast intrinsic protein	7.532183671	185.1028978	6.35E-06
Zm.19048.1.A1_at	Sorbitol transporter	5.545369448	46.70060867	5.97E-07
Zm.12135.1.A1_at	Cysteine protease 1 precursor	4.820477483	28.25584605	3.58E-05
Zm.12789.1.A1_at	Cytochrome P450	4.228409653	18.74468469	5.52E-07
Zm.17956.1.A1_at	Ethylene responsive element binding factor 5	4.14001278	17.63063803	4.22E-09
Zm.280.1.A1_at	luminal binding protein	4.118441629	17.36898603	2.67E-07
Zm.13983.1.S1_at	Embryo-specific protein	3.972934156	15.70262835	4.22E-06
Zm.13506.1.S1_at	Seed maturation protein	3.894397187	14.87066426	2.82E-06
Zm.395.1.S1_at	Luminal binding protein	3.795452084	13.88496932	6.39E-08
Zm.2671.1.S1_at	Transposon protein en spm sub-class	3.616637828	12.26638156	0.000137
Zm.17912.1.S1_at	Inositol phosphorylceramide synthase1	3.598109682	12.10985497	2.51E-05
Zm.9422.1.A1_at	WRKY transcription factor 24	3.40291766	10.57743313	3.97E-05
Zm.4089.2.S1_at	Hexose transporter	3.066212285	8.375714608	1.62E-05

Table S2 (continued).

Probe ID	Sequence description	log FC	Absolute FC	*p* value
Zm.2793.1.A1_at	Aldehyde dehydrogenase family 2 member mitochondrial-like	2.987241482	7.929563666	0.000106
Zm.16211.1.S1_at	Cell division protein aaa ATPase family	2.805479082	6.990904283	5.42E-08
Zm.12825.1.S1_at	Tonoplast intrinsic protein	2.783585218	6.885613572	0.000197
Zm.54.1.S1_at	Alpha amylase isozyme 3e precursor	2.630964856	6.194401324	4.2E-06
Zm.8865.1.A1_at	Integral membrane protein	2.551747452	5.863440526	1.55E-05
Zm.12796.1.A1_at	Uncharacterized conserved expressed	2.547823296	5.847513537	1.24E-05
Zm.15061.1.S1_at	Calmodulin-like protein 14	2.496638576	5.64368934	4.71E-06
Zm.12327.1.A1_at	Heat shock transcription factor	2.98886809	7.938509121	1.64E-08

Supplementary Table S3. Transcription factors unique to HKI1532 and their expression values under water stress.

Probe ID	TF	Sequence description	log FC	Absolute FC
Zm.15542.1.S1_at	ARF	IAA9-auxin responsive aux iaa family member	2.666122	6.347206295
Zm.6938.1.A1_at	bHLH		2.85758	7.247982958
Zm.10293.1.S1_at	bZIP	Light inducible protein cprf-2	2.828891	7.105276351
Zm.3849.1.S1_at	bZIP	Glutamate decarboxylase	4.095848	17.0990915
Zm.16522.1.S1_at	C3H	Kh domain-containing protein	2.379782	5.204579588
Zm.5544.1.A1_at	C3H	Zinc finger ccch domain-containing protein 11-like	2.430087	5.3892598
Zm.17956.1.A1_at	ERF	Ethylene responsive element binding factor 5	4.140013	17.63063803
Zm.7368.1.S1_at	ERF	Dehydration-responsive element binding protein 2c	2.835316	7.136992221
Zm.14048.1.A1_at	GRAS	Stearoyl-acyl-carrier protein desaturase	2.93234	7.633474837
Zm.12928.1.A1_at	HB-other	E3- ubiquitin protein ligase ring1-like	2.978913	7.883918966
Zm.16320.1.S1_at	HB-other	Protein binding protein	3.672082	12.74696862
Zm.5362.1.A1_at	HB-other	Rop guanine nucleotide exchange factor	2.981591	7.898568637
Zm.4514.1.A1_at	HD-ZIP	Homeodomain leucine zipper family IV protein	2.752628	6.73943558

Zm.10032.1.S1_at	HSF	Heat shock factor protein 4	3.452248	10.94536587
Zm.12327.1.A1_at	HSF	Protein	2.988868	7.938509121
Zm.15325.1.A1_at	HSF	Heat shock factor protein hsf30	2.68685	6.439059398
Zm.15688.1.S1_at	HSF		5.270073	38.58779506
Zm.11950.1.A1_at	LBD	Receptor like protein kinase hsl1-like	2.951758	7.736914835
Zm.13464.1.S1_at	LBD	ATP binding protein	3.685086	12.86237845
Zm.13947.3.A1_at	LBD	cipk like protein expressed	2.738064	6.671742128
Zm.13996.1.S1_at	LBD	Serine threonine protein kinase ht1-like	3.256065	9.55373685
Zm.243.1.A1_at	LBD	g-type lectin s-receptor like serine threonine protein kinase	2.979453	7.886871182
Zm.6021.1.A1_at	LBD	Receptor protein	2.570086	5.938449094
Zm.721.1.A1_at	LBD	Protein kinase pti1	2.555573	5.879009144
Zm.11627.1.A1_x_at	MIKC	mads box transcription factor 26	3.177698	9.048621014
Zm.8773.1.A1_at	MYB	Uncharacterized protein	5.167537	35.94045447
Zm.10778.1.A1_a_at	MYB_related	Glabura2 expression modulator	3.049708	8.280444176
Zm.18735.1.A1_at	MYB_related	Ribonucleoprotein like protein	2.537344	5.805194862
Zm.4251.1.S1_at	MYB_related	Protein disulfide	3.249836	9.512578603

		isomerase		
Zm.4558.1.S1_at	MYB_related	Ectonucleotide pyrophosphatase phosphodiesterase family member	2.75114	6.732487099
Zm.4967.1.A1_at	MYB_related	Telomere repeat binding protein 5-like	2.834515	7.13303164
Zm.7870.1.S1_at	MYB_related	Protein	4.405614	21.19444524
Zm.10304.1.S1_at	NAC	4 protein	3.263138	9.60069243
Zm.12113.1.A1_at	NAC		2.430397	5.390417005
Zm.5882.1.A1_at	NAC	4 protein	3.850999	14.42999231
Zm.13736.1.A1_at	NF-X1	nf-x1-type zinc finger protein	4.153184	17.7923362
ZmAffx.1300.1.S1_at	SBP		3.850849	14.42849441
Zm.17900.1.S1_at	TCP	Chaperone protein dnaj	3.138252	8.804565395
Zm.5083.1.A1_a_at	TCP	dnaj protein homolog	4.425882	21.49430364
Zm.5083.2.A1_x_at	TCP	dnaj protein homolog	7.830876	227.6819593
Zm.5083.3.A1_at	TCP	dnaj protein homolog	7.445254	174.2788527
Zm.10017.1.A1_at	WRKY	Wrky69-superfamily of tfs having wrky and zinc finger domains	3.299544	9.846044689
Zm.9422.1.A1_at	WRKY		3.402918	10.57743313
Zm.8827.1.A1_at	YABBY		3.153776	8.899821159

Supplementary Table S4. A set of 174 selected drought-responsive genes from HKI1532 and their co-expression network groups.

Probe ID	Gene	Gene symbol used in Coexpression Network	Absolute FC	Regulation	Group
Zm.10017.1.A1_at	WRKY	WRKY	9.8460	up	G1
Zm.10304.1.S1_at	NAC	NAC	9.6007	up	G1
Zm.12113.1.A1_at	NAC	NAC	5.3904	up	G1
Zm.12327.1.A1_at	HSF	HSF	7.9385	up	G1
Zm.16211.1.S1_at	ERF	ERF	6.9909	up	G1
Zm.17956.1.A1_at	AP2	AP2	17.6306	up	G1
Zm.18735.1.A1_at	MYB_related	MYB_related	5.8052	up	G1
Zm.4558.1.S1_at	MYB_related	MYB_related	6.7325	up	G1
Zm.5112.1.A1_at	atpase subunit 8	ATPase	7.9600	up	G1
Zm.5882.1.A1_at	NAC	NAC	14.4300	up	G1
Zm.7368.1.S1_at	AP2	AP2	7.1370	up	G1
Zm.7870.1.S1_at	MYB_related	MYB_related	21.1944	up	G1
Zm.8773.1.A1_at	MYB	MYB	35.9405	up	G1
Zm.8865.1.A1_at	WRKY	WRKY	5.8634	up	G1
Zm.9422.1.A1_at	WRKY	WRKY	10.5774	up	G1
ZmAffx.1219.1.S1_s_at	atp synthase subunit alpha	ATP_synthase	8.7593	up	G1
ZmAffx.1300.1.S1_at	SBP	SBP	14.4285	up	G1
ZmAffx.1401.1.S1_at	atpase subunit 8	ATPase	22.6600	up	G1
ZmAffx.1474.1.S1_s_at	atpase subunit 9	ATPase	18.6416	up	G1
Zm.10383.4.S1_a_at	NF-YB	NF-YB	5.6872	up	G1
Zm.11645.1.A1_at	ring-h2 zinc finger protein atl6-like	Ring_H2_ZnFP	16.5903	up	G1
Zm.11783.1.A1_at	MYB_related	MYB_related	25.2762	up	G1
Zm.13603.1.S1_at	WRKY	WRKY	10.8794	up	G1
Zm.13801.1.S1_at	ERF	ERF	6.0215	up	G1
Zm.2629.1.S1_at	bZIP	bZIP	9.5268	up	G1
Zm.2807.1.A1_at	zf-C2H2_6	C2H2	16.8529	up	G1
Zm.4838.1.A1_at	NAC	NAC	10.5986	up	G1
Zm.5212.1.S1_at	ERF	ERF	5.0598	up	G1
Zm.5979.1.A1_at	MYB_related	MYB_related	22.6368	up	G1
Zm.6938.1.A1_at	bHLH	bHLH	7.2480	up	G1
Zm.7780.1.A1_at	abscisic acid 8 -hydroxylase	ABA-8H	9.1029	up	G1

Table S4 (continued).

Probe ID	Gene	Gene symbol used in Coexpression Network	Absolute FC	Regulation	Group
Zm.8972.1.S1_at	AP2	AP2	136.1449	up	G1
Zm.9463.1.A1_at	ERF	ERF	31.6441	up	G1
Zm.9645.1.S1_at	MYB_related	MYB_related	5.0466	up	G1
Zm.9930.1.A1_at	Similar to Abscisic stress ripening protein 1	ASR_protein	31.2614	up	G1
Zm.11848.1.A1_at	ef hand family protein	EF_hand_4	29.6250	up	G2
Zm.11950.1.A1_at	Pkinase	Pkinase	7.7369	up	G2
Zm.12648.1.A1_at	calcium-dependent protein kinase	CDPK	6.8351	up	G2
Zm.13464.1.S1_at	Pkinase	Pkinase	12.8624	up	G2
Zm.13947.3.A1_at	Pkinase	Pkinase	6.6717	up	G2
Zm.13996.1.S1_at	Pkinase_Tyr	Pkinase_Tyr	9.5537	up	G2
Zm.15061.1.S1_at	efhand	efhand	5.6437	up	G2
Zm.168.1.A1_at	receptor-like protein kinase	Pkinase	11.1913	up	G2
Zm.17842.1.S1_at	EF_hand_5	EF_hand_5	5.0894	up	G2
Zm.5761.1.A1_at	EF_hand_5	EF_hand_5	13.1971	up	G2
Zm.6021.1.A1_at	Pkinase	Pkinase	5.9384	up	G2
Zm.721.1.A1_at	protein kinase pti1	protein_kinase	5.8790	up	G2
Zm.9675.1.A1_at	signalling.G-proteins	G-proteins	16.5283	up	G2
Zm.10267.1.A1_at	Similar to Calmodulin-like protein	Calmodulin	44.6102	up	G2
Zm.10480.1.S1_at	EF_hand_5	EF_hand_5	7.6222	up	G2
Zm.1162.1.S1_at	ef-hand calcium binding	Efhand_Ca	10.5453	up	G2
Zm.11745.1.A1_at	mitogen-activated protein kinase kinase kinase a-like	MAPKKK-like	41.5661	up	G2
Zm.12139.1.A1_at	calcium binding protein with ef-hand motif	Efhand_Ca	23.9415	up	G2
Zm.1442.2.S1_at	EF_hand_5	EF_hand_5	6.3463	up	G2

Table S4 (continued).

Probe ID	Gene	Gene symbol used in Coexpression Network	Absolute FC	Regulation	Group
Zm.15379.1.A1_at	mitogen-activated protein kinase-like	MAPK	6.4221	up	G2
Zm.17723.1.S1_at	EF_hand_3	EF_hand_3	10.6374	up	G2
Zm.352.1.S1_at	calmodulin	calmodulin	12.5478	up	G2
Zm.5036.1.A1_at	protein kinase	protein_kinase	7.8306	up	G2
Zm.5299.1.S1_at	protein_kinase	protein_kinase	9.0731	up	G2
Zm.6367.1.S1_at	acc synthase	ACC_synthase	26.9101	up	G2
ZmAffx.777.1.A1_at	serine threonine-specific protein kinase npk15-like	Pkinase_Tyr	5.7526	up	G2
Zm.12842.1.A1_at	glycolate oxidase	glycolate_oxydase	-10.2520	down	G3
Zm.1332.1.S1_at	atp synthase protein i	ATP_synthase	-11.5518	down	G3
Zm.16683.1.S1_at	chlorophyll a b-binding protein type ii	chl_A-B_BP	-5.3993	down	G3
Zm.2601.1.S1_at	glyceraldehyde-3-phosphate	G3PD	10.3605	up	G3
Zm.3449.1.S1_at	atp synthase delta chain	ATP_synthase	-6.1518	down	G3
Zm.3895.2.A1_at	PS.lightreaction.photosystem II. PSII polypeptide subunits	PSII	-6.4430	down	G3
Zm.5828.1.A1_at	phosphoglycerate kinase	PGK	-7.9285	down	G3
Zm.6763.1.S1_at	ribulose- -bisphosphate carboxylase oxygenase small subunit	RuBisCo	-6.3449	down	G3
Zm.7091.1.A1_at	chlorophyll a-b binding protein cp24	chl_A-B_BP	-5.8528	down	G3

Table S4 (continued).

Probe ID	Gene	Gene symbol used in Coexpression Network	Absolute FC	Regulation	Group
Zm.10401.1.S1_at	PS.photorespiration. glycolate oxydase	glycolate_oxydase	-7.9978	down	G3
Zm.1088.1.S1_at	atp synthase gamma chain	ATP_synthase	-85.9131	down	G3
Zm.13239.2.A1_at	chlorophyll a-b binding protein 2	chl_A-B_BP	-6.3594	down	G3
Zm.18297.1.S1_at	fructose- - bisphosphatase	FBPase	-90.5095	down	G3
Zm.12134.3.A1_a_at	granule-bound starch synthase	Stach_synthase	7.7288	up	G4
Zm.18317.1.A1_at	sucrose phosphate synthase	SPS	8.7263	up	G4
Zm.54.1.S1_at	alpha-amylase isozyme 3e precursor	Alpha-amylase	6.1944	up	G4
Zm.299.1.S1_a_at	malate synthase	malate_synthase	11.1339	up	G4
Zm.3762.1.A1_at	beta-amylase	beta-amylase	63.8023	up	G4
Zm.3883.1.S1_at	beta-amylase	beta-amylase	10.7594	up	G4
Zm.409.1.S1_at	cell wall invertase	invertase	13.0407	up	G4
Zm.8365.1.A1_at	hexokinase 1	hexokinase_1	7.6251	up	G4
Zm.12825.1.S1_at	tonoplast intrinsic protein	TIP3-1	6.8856	up	G5
Zm.171.1.A2_at	voltage-dependent anion channel	VDAC	-13.1264	down	G5
Zm.622.1.S1_at	tonoplast intrinsic protein	TIP	185.1029	up	G5
Zm.13050.1.A1_at	integral membrane single c2 domain protein	IMP	6.7528	up	G5
Zm.2959.1.S1_at	AWPM-19	AWPM-19	136.2445	up	G5
Zm.3906.1.A1_at	na+ h+ antiporter	Na+_H+_antiporter	19.0454	up	G5

Table S4 (continued).

Probe ID	Gene	Gene symbol used in Coexpression Network	Absolute FC	Regulation	Group
Zm.43.1.S1_a_at	Similar to Isoform 2 of Late embryogenesis abundant protein	LEA	1315.5604	up	G5
Zm.6095.1.S1_at	vacuolar proton-inorganic pyrophosphatase	H_PPase	9.5673	up	G5
Zm.616.1.S1_at	tonoplast intrinsic protein	TIP	4818.6917	up	G5
Zm.10032.1.S1_at	HSF	HSF	10.9454	up	G6
Zm.13506.1.S1_at	Late embryogenesis abundant (LEA) group 1 family protein	LEA	14.8707	up	G6
Zm.15325.1.A1_at	HSF	HSF	6.4391	up	G6
Zm.16525.1.A1_at	heat shock protein 90	HSP90	19.4948	up	G6
Zm.4251.1.S1_at	protein disulfide isomerase	PDI	9.5126	up	G6
Zm.4273.2.A1_at	heat shock 70 kda protein 4	HSP70	11.8497	up	G6
Zm.5048.6.A1_a_at	heat shock protein 90	HSP90	5.1986	up	G6
Zm.5970.1.S1_at	subtilisin-like protease	subtilisin_protease	5.0545	up	G6
Zm.6353.1.S1_at	heat shock protein sti-like	Hsp	8.1336	up	G6
Zm.10095.1.A1_at	hsp20-like chaperone	Chaperone	8.6478	up	G6
Zm.12227.1.S1_at	heat shock cognate 70 kda protein 2	HSP70	439.1870	up	G6
Zm.12517.1.A1_at	HSP20	HSP20	777.1541	up	G6
Zm.15462.1.S1_at	heat-shock protein 70t-2	HSP70	605.6317	up	G6
Zm.16504.1.A1_at	kda class ii heat shock protein	classII_Hsp	164.2669	up	G6
Zm.16504.2.A1_at	kda class ii heat shock protein	classII_Hsp	608.5566	up	G6

Table S4 (continued).

Probe ID	Gene	Gene symbol used in Coexpression Network	Absolute FC	Regulation	Group
Zm.16505.1.A1_at	heat shock protein 90	HSP90	8.9312	up	G6
Zm.16505.3.A1_at	heat shock protein 90	HSP90	15.6185	up	G6
Zm.16525.2.S1_a_at	heat shock protein 90	HSP90	170.3164	up	G6
Zm.16959.2.A1_at	kda class i heat shock	ClassI_Hsp	285.0497	up	G6
Zm.1882.1.S1_at	late embryogenesis abundant protein	LEA	6.2180	up	G6
Zm.191.1.A1_at	heat shock protein 70	HSP70	416.5075	up	G6
Zm.2361.1.A1_at	kda class i heat shock protein 1	ClassI_Hsp	6541.7460	up	G6
Zm.2482.1.A1_at	kda class i heat shock protein 1	ClassI_Hsp	67.1456	up	G6
Zm.2497.1.S1_at	heat shock protein	Hsp	120.9187	up	G6
Zm.311.1.A1_at	kda class ii heat shock protein	classII_Hsp	248.3084	up	G6
Zm.369.1.A1_at	heat-shock protein 20	HSP20	1994.3936	up	G6
Zm.3693.1.A1_at	kda class i heat shock protein 1	Class_I_Hsp	1868.7272	up	G6
Zm.3740.1.A1_at	kda class iv heat shock protein precursor	classIV_Hsp	976.0863	up	G6
Zm.438.1.S1_at	heat shock protein 101	Hsp	635.6821	up	G6
Zm.7900.1.A1_at	atp-dependent clp protease atp-binding subunit precursor	ATP-clp_protease	10.6947	up	G6
Zm.11660.1.A1_at	acetyl- synthetase	Acyl_CoA_ligase	11.4370	up	G7
Zm.12135.1.A1_at	Peptidase_C1	Peptidase_C1	28.2558	up	G7
Zm.12789.1.A1_at	cytochrome p450	p450	18.7447	up	G7

Table S4 (continued).

Probe ID	Gene	Gene symbol used in Coexpression Network	Absolute FC	Regulation	Group
Zm.17912.1.S1_at	inositol phosphorylceramide synthase 1	PAP-like	12.1099	up	G7
Zm.2671.1.S1_at	Phospholipase A2, active site domain containing protein	Phospholipase_A2	12.2664	up	G7
Zm.4877.1.S1_at	beta-ketoacyl-acp synthase	ACP_synthase	-5.3292	down	G7
Zm.7407.1.A1_a_at	squalene monooxygenase	squalene_MO	8.1116	up	G7
Zm.11770.1.A1_at	Phospholipase A2, active site domain containing protein	Phospholipase_A2	6.2287	up	G7
Zm.12107.1.A1_at	cytochrome p450	p450	32.4015	up	G7
Zm.13167.1.A1_at	phosphatidylserine synthase 2	PSS	6.5494	up	G7
Zm.14226.1.A1_at	cytochrome p450	p450	59.8887	up	G7
Zm.15532.1.S1_at	cytochrome p450 89a2-like	p450	17.5465	up	G7
Zm.15540.1.S1_at	Phospholipase D alpha 1	PhospholipaseD	7.6308	up	G7
Zm.16251.1.A1_at	protein phosphatase 2c abi1	PP2C	57.5343	up	G7
Zm.17264.1.A1_a_at	cytochrome p450 81d1-like	p450	29.2600	up	G7
Zm.19112.1.A1_at	cytochrome p450	p450	20.1450	up	G7
Zm.2032.1.S1_at	cytochrome p450	p450	11.0156	up	G7
Zm.445.1.S1_at	lipoxygenase 1	LOX1	11.0874	up	G7
Zm.15542.1.S1_at	iaa9 - auxin-responsive aux iaa family member	Aux_IAA	6.3472	up	G8

Table S4 (continued).

**Probe ID**	**Gene**	**Gene symbol used in Coexpression Network**	**Absolute FC**	**Regulation**	**Group**
Zm.1616.1.A1_at	Similar to Peroxidase BP 1 precursor	peroxidase1-like	-8.7304	down	G8
Zm.17905.1.S1_at	flavin-containing monooxygenase fmo gs-ox-like 3-like	FMO_like	6.2131	up	G8
Zm.10676.1.S1_at	12-oxophytodienoic acid reductase	12-OPR	267.2536	up	G8
Zm.14498.2.A1_at	expansin a1	expansin_a1	15.9133	up	G8
Zm.1527.2.A1_a_at	xyloglucan endotransglucosylase hydrolase protein 23 precursor	XET_hydrolase	32.0586	up	G8
Zm.15456.1.A1_at	xyloglucan galactosyltransferase katamari1-like	XET_katamari1	8.8858	up	G8
Zm.704.1.S1_at	probable xyloglucan endotransglucosylase hydrolase protein 23	XET_hydrolase	28.2553	up	G8
Zm.8927.1.S1_at	cell wall.cell wall proteins.AGPs	AGPs	129.0533	up	G8
Zm.9297.1.A1_at	cell wall.cell wall proteins.AGPs	AGPs	64.6181	up	G8
Zm.13609.1.S1_at	tonoplast dicarboxylate transporter-like	TDT	7.7288	up	G9
Zm.14368.1.A1_at	potassium transporter hak2p	K_transporter	11.4091	up	G9
Zm.19048.1.A1_at	sorbitol transporter	Sugar_transporter	46.7006	up	G9

Table S4 (continued).

Probe ID	Gene	Gene symbol used in Coexpression Network	Absolute FC	Regulation	Group
Zm.10826.1.S1_at	vacuolar amino acid transporter 1-like	AVT1	5.2827	up	G9
Zm.13688.1.S1_at	Similar to MtN3 protein precursor	MtN3-like	695.3546	up	G9
Zm.16478.3.A1_a_at	abc transporter c family member 8-like	ABC_transporter	55.6952	up	G9
Zm.19239.1.A1_at	carbohydrate transporter sugar porter transporter	CHO_transporter	17.5054	up	G9
Zm.4089.1.S1_at	hexose transporter	hexose_transporter	8.8766	up	G9
Zm.7545.1.A1_at	sugar transporter	sugar_transporter	10.2086	up	G9
Zm.9995.1.A1_at	MtN3_slv	MtN3	6.2435	up	G9
Zm.107.1.S1_at	glutamine synthetase	Gln-synt_C	155.4548	up	G10
Zm.16478.2.S1_at	aspartate aminotransferase	AspAT	16.4664	up	G10
Zm.17181.1.S1_at	pyridoxal phosphate-dependent enzyme domain containing protein	PyrdxlP-dep_enz	40.6412	up	G10
Zm.17982.1.A1_at	Amino_oxidase	Amino_oxidase	9.3929	up	G10
Zm.5534.1.S1_at	s-adenosylmethionine decarboxylase	SAM_decarbox	7.0383	up	G10
Zm.246.1.A1_at	glutathione s-transferase	GST	15.1098	up	G11
Zm.2793.1.A1_at	aldehyde dehydrogenase family 2	ADH	7.9296	up	G11
Zm.562.1.A1_at	glutathione s-transferase gstu6	GST	9.1296	up	G11

Table S4 (continued).

Probe ID	Gene	Gene symbol used in Coexpression Network	Absolute FC	Regulation	Group
Zm.5873.1.S1_at	glutathione s-transferase	GST	7.4667	up	G11
Zm.627.1.A1_at	glutathione s-transferase	GST	9.4673	up	G11
Zm.13591.1.S1_at	probable glutathione s-transferase gstu6-like	GST	8.0440	up	G11
Zm.1987.2.A1_at	Superoxide dismutase-3 precursor	SOD	22.6019	up	G11
Zm.2193.1.A1_at	glutathione s-transferase gstu6	GST	58.1546	up	G11
Zm.3996.1.S1_at	gdp-mannose pyrophosphorylase	GMPase	17.1766	up	G11
Zm.6203.2.A1_a_at	phospholipid hydroperoxide glutathione peroxidase	GPx	8.4774	up	G11

Supplementary Table S5. Statistical parameters of the co-expression network of genes specific to drought tolerance.

Probe ID	Average shortest pathlength	Clustering coefficient	Closeness centrality	Degree	Neighborhood connectivity
Zm.12134.3.A1_a_at	2.4765	0.6579	0.4038	20	32.4500
Zm.12789.1.A1_at	2.9588	0.7619	0.3380	7	17.5714
Zm.12825.1.S1_at	2.5294	0.6667	0.3953	15	30.0000
Zm.12842.1.A1_at	3.2059	0.9048	0.3119	7	16.7143
Zm.16251.1.A1_at	2.3588	0.5810	0.4239	23	30.7826
Zm.721.1.A1_at	3.0471	0.7143	0.3282	8	15.5000
Zm.9995.1.A1_at	0.0000	0.0000	0.0000	0	0.0000
Zm.10304.1.S1_at	2.7765	0.6800	0.3602	25	23.6400
Zm.10826.1.S1_at	3.2706	0.6667	0.3058	3	8.6667
Zm.1162.1.S1_at	3.4235	0.8889	0.2921	10	24.4000
Zm.11645.1.A1_at	2.5765	0.2924	0.3881	43	16.1163
Zm.11783.1.A1_at	2.8294	0.6341	0.3534	24	21.7917
Zm.12107.1.A1_at	2.7412	0.7574	0.3648	17	26.3529
Zm.12113.1.A1_at	2.8588	0.7333	0.3498	15	26.3333
Zm.13591.1.S1_at	2.8118	0.5000	0.3556	4	25.2500
Zm.13609.1.S1_at	3.2706	1.0000	0.3058	3	29.6667
Zm.168.1.A1_at	2.7941	0.5217	0.3579	24	20.0833
Zm.17723.1.S1_at	2.7176	0.5810	0.3680	23	22.0000
Zm.2032.1.S1_at	3.9353	0.0000	0.2541	1	17.0000
Zm.2671.1.S1_at	3.1059	0.6429	0.3220	8	27.2500
Zm.3762.1.A1_at	3.2765	0.6667	0.3052	3	13.3333
Zm.5112.1.A1_at	3.2235	0.5357	0.3102	8	11.7500
Zm.54.1.S1_at	3.2529	0.6667	0.3074	4	24.5000
Zm.6938.1.A1_at	2.9412	0.5074	0.3400	17	17.7059
Zm.7368.1.S1_at	3.6412	1.0000	0.2746	3	27.0000
Zm.7780.1.A1_at	3.3824	0.6667	0.2957	3	14.3333
Zm.9422.1.A1_at	2.7059	0.4397	0.3696	36	19.3889
Zm.9930.1.A1_at	2.8471	0.8129	0.3512	19	26.0000
ZmAffx.1219.1.S1_s_at	2.6824	0.5714	0.3728	7	18.5714
ZmAffx.1300.1.S1_at	2.5353	0.1897	0.3944	23	11.7826
ZmAffx.1401.1.S1_at	3.2412	1.0000	0.3085	5	17.2000
ZmAffx.1474.1.S1_s_at	3.2412	1.0000	0.3085	5	17.2000
Zm.12135.1.A1_at	2.3765	0.6739	0.4208	24	36.4167
Zm.12327.1.A1_at	3.8294	1.0000	0.2611	3	22.0000
Zm.12648.1.A1_at	3.2824	0.8000	0.3047	5	20.2000
Zm.13464.1.S1_at	2.9824	0.7524	0.3353	15	21.9333

Table S5 (continued).

Probe ID	Average shortest pathlength	Clustering coefficient	Closeness centrality	Degree	Neighborhood connectivity
Zm.13688.1.S1_at	3.1706	1.0000	0.3154	3	33.3333
Zm.13996.1.S1_at	2.2294	0.4115	0.4485	40	27.5250
Zm.14368.1.A1_at	2.2765	0.5161	0.4393	32	31.4375
Zm.14498.2.A1_at	2.3706	0.4577	0.4218	28	23.1429
Zm.1527.2.A1_a_at	3.0000	0.9697	0.3333	12	25.0833
Zm.15540.1.S1_at	3.4824	1.0000	0.2872	2	16.5000
Zm.16211.1.S1_at	2.8647	0.7424	0.3491	12	25.0000
Zm.17264.1.A1_a_at	3.2059	1.0000	0.3119	2	29.0000
Zm.2193.1.A1_at	0.0000	0.0000	0.0000	0	0.0000
Zm.2601.1.S1_at	2.4118	0.6087	0.4146	23	27.4348
Zm.3996.1.S1_at	2.4294	0.6877	0.4116	23	36.3043
Zm.5036.1.A1_at	3.0176	0.9000	0.3314	5	29.2000
Zm.5212.1.S1_at	2.3118	0.5291	0.4326	28	29.6429
Zm.5299.1.S1_at	2.8176	0.7574	0.3549	17	24.5882
Zm.5970.1.S1_at	2.9412	0.5667	0.3400	21	19.9524
Zm.6021.1.A1_at	2.8824	0.4308	0.3469	23	18.1739
Zm.6095.1.S1_at	2.5000	0.6209	0.4000	18	24.6111
Zm.7407.1.A1_a_at	2.9294	0.5628	0.3414	22	19.2273
Zm.10095.1.A1_at	2.9353	0.8333	0.3407	9	16.3333
Zm.10676.1.S1_at	2.8471	0.8363	0.3512	19	26.2632
Zm.13506.1.S1_at	2.3941	0.5163	0.4177	18	23.7222
Zm.13603.1.S1_at	3.4588	1.0000	0.2891	3	38.3333
Zm.13801.1.S1_at	3.2765	1.0000	0.3052	3	16.3333
Zm.15542.1.S1_at	2.9941	0.7143	0.3340	8	25.6250
Zm.17181.1.S1_at	3.2588	1.0000	0.3069	4	19.0000
Zm.17912.1.S1_at	2.7294	0.5101	0.3664	32	20.7188
Zm.17956.1.A1_at	2.9059	0.9286	0.3441	8	29.1250
Zm.18735.1.A1_at	2.6706	0.4762	0.3744	7	15.8571
Zm.19112.1.A1_at	2.8235	0.5628	0.3542	22	20.6364
Zm.1987.2.A1_at	2.6529	0.4396	0.3769	14	13.0714
Zm.2959.1.S1_at	2.3000	0.4762	0.4348	21	26.0476
Zm.299.1.S1_a_at	2.5706	0.9273	0.3890	11	49.2727
Zm.43.1.S1_a_at	2.7824	0.5000	0.3594	12	12.8333
Zm.445.1.S1_at	2.8765	0.8485	0.3476	12	27.3333
Zm.5534.1.S1_at	3.9353	0.0000	0.2541	1	17.0000
Zm.562.1.A1_at	3.1353	0.6667	0.3189	3	26.0000

Table S5 (Continued).

Probe ID	Average shortest pathlength	Clustering coefficient	Closeness centrality	Degree	Neighborhood connectivity
Zm.5882.1.A1_at	2.7529	0.5862	0.3632	29	21.8621
Zm.616.1.S1_at	2.4000	0.5229	0.4167	18	22.6667
Zm.622.1.S1_at	2.3941	0.5029	0.4177	19	23.4211
Zm.7900.1.A1_at	2.8176	0.7190	0.3549	21	25.0000
Zm.9297.1.A1_at	2.6706	0.3846	0.3744	40	18.2750
Zm.10017.1.A1_at	2.8118	0.7101	0.3556	24	24.2500
Zm.11770.1.A1_at	2.7588	0.6571	0.3625	21	23.2857
Zm.14226.1.A1_at	2.8941	0.7576	0.3455	12	24.7500
Zm.6367.1.S1_at	2.8588	0.8476	0.3498	15	26.8667
Zm.9675.1.A1_at	3.0000	1.0000	0.3333	6	22.6667
Zm.1442.2.S1_at	2.9176	0.6813	0.3427	14	23.0000
Zm.15379.1.A1_at	4.4059	0.0000	0.2270	1	10.0000
Zm.2629.1.S1_at	2.4529	0.6397	0.4077	17	35.1765
Zm.352.1.S1_at	2.7882	0.6209	0.3586	18	22.5556
Zm.5761.1.A1_at	3.4118	0.5333	0.2931	10	14.5000
Zm.6203.2.A1_a_at	3.6353	1.0000	0.2751	3	32.6667
Zm.8865.1.A1_at	2.9294	0.3571	0.3414	8	15.2500
Zm.12517.1.A1_at	2.9000	0.7895	0.3448	19	19.0000
Zm.15462.1.S1_at	2.7118	0.5671	0.3688	22	18.5909
Zm.16504.1.A1_at	2.8529	0.8562	0.3505	18	20.5000
Zm.16504.2.A1_at	2.9118	0.9118	0.3434	17	20.1176
Zm.16505.3.A1_at	2.7941	0.7310	0.3579	19	22.0000
Zm.16525.1.A1_at	3.0647	0.5909	0.3263	12	18.0833
Zm.16525.2.S1_a_at	2.5000	0.4091	0.4000	12	36.8333
Zm.16959.2.A1_at	2.8471	0.8070	0.3512	19	19.8421
Zm.191.1.A1_at	2.3353	0.4933	0.4282	25	32.4000
Zm.2361.1.A1_at	2.9118	0.9118	0.3434	17	20.1176
Zm.2497.1.S1_at	2.9000	0.7895	0.3448	19	19.0000
Zm.311.1.A1_at	2.9118	0.9118	0.3434	17	20.1176
Zm.369.1.A1_at	2.8176	0.6952	0.3549	21	19.5238
Zm.3693.1.A1_at	2.8882	0.5417	0.3462	16	17.5625
Zm.3740.1.A1_at	3.4765	1.0000	0.2876	12	19.2500
Zm.4273.2.A1_at	2.8588	0.5931	0.3498	22	17.5909
Zm.438.1.S1_at	2.8235	0.7158	0.3542	20	19.6000
Zm.5873.1.S1_at	3.6059	0.8611	0.2773	9	19.7778
Zm.5979.1.A1_at	3.6647	1.0000	0.2729	2	21.5000

Table S5 (Continued).

Probe ID	Average shortest pathlength	Clustering coefficient	Closeness centrality	Degree	Neighborhood connectivity
Zm.6353.1.S1_at	2.8353	0.4929	0.3527	27	21.0741
Zm.7870.1.S1_at	2.9059	0.7053	0.3441	20	18.5500
Zm.10032.1.S1_at	2.2765	0.5931	0.4393	22	30.8182
Zm.10267.1.A1_at	2.7118	0.5394	0.3688	29	21.0345
Zm.10401.1.S1_at	2.6176	0.9500	0.3820	16	40.3125
Zm.10480.1.S1_at	2.9176	0.8030	0.3427	12	23.5833
Zm.107.1.S1_at	2.7059	1.0000	0.3696	8	53.5000
Zm.1088.1.S1_at	1.8353	0.3091	0.5449	56	23.0357
Zm.11660.1.A1_at	2.7000	1.0000	0.3704	9	52.4444
Zm.11745.1.A1_at	2.4588	0.5000	0.4067	4	43.5000
Zm.11848.1.A1_at	2.9471	0.8667	0.3393	10	23.2000
Zm.11950.1.A1_at	2.3529	0.6397	0.4250	17	34.1765
Zm.12139.1.A1_at	2.3706	0.5556	0.4218	18	38.1111
Zm.12227.1.S1_at	2.6353	1.0000	0.3795	13	46.1538
Zm.13050.1.A1_at	2.7824	1.0000	0.3594	2	57.5000
Zm.13167.1.A1_at	2.7353	1.0000	0.3656	6	54.8333
Zm.13239.2.A1_at	1.9941	0.4061	0.5015	47	25.0000
Zm.1332.1.S1_at	1.8529	0.2887	0.5397	59	22.0000
Zm.13947.3.A1_at	2.5882	0.9231	0.3864	14	42.9286
Zm.15325.1.A1_at	3.8294	1.0000	0.2611	3	22.0000
Zm.15456.1.A1_at	2.2824	0.5365	0.4381	34	29.5588
Zm.15532.1.S1_at	2.2353	0.3942	0.4474	28	26.8214
Zm.1616.1.A1_at	2.4824	0.7879	0.4028	22	34.3182
Zm.16478.2.S1_at	2.3941	0.4815	0.4177	27	28.5556
Zm.16478.3.A1_a_at	2.9647	0.8095	0.3373	7	21.4286
Zm.16683.1.S1_at	1.9294	0.3529	0.5183	49	23.7959
Zm.171.1.A2_at	2.7353	1.0000	0.3656	6	54.8333
Zm.17842.1.S1_at	2.3471	0.5556	0.4261	19	37.6842
Zm.17905.1.S1_at	2.6882	1.0000	0.3720	11	49.4545
Zm.17982.1.A1_at	2.6941	1.0000	0.3712	10	52.1000
Zm.18297.1.S1_at	1.8235	0.3156	0.5484	56	23.0000
Zm.18317.1.A1_at	2.7059	1.0000	0.3696	8	53.5000
Zm.1882.1.S1_at	2.6412	1.0000	0.3786	12	48.1667
Zm.19048.1.A1_at	2.8000	1.0000	0.3571	4	53.5000
Zm.19239.1.A1_at	2.4118	0.6444	0.4146	10	34.0000
Zm.246.1.A1_at	2.6353	1.0000	0.3795	13	45.6923

Table S5 (Continued).

Probe ID	Average shortest pathlength	Clustering coefficient	Closeness centrality	Degree	Neighborhood connectivity
Zm.2482.1.A1_at	2.6824	1.0000	0.3728	12	47.1667
Zm.2793.1.A1_at	2.4294	0.8789	0.4116	20	38.9500
Zm.2807.1.A1_at	2.8647	0.6947	0.3491	20	22.3000
Zm.3449.1.S1_at	1.8706	0.3235	0.5346	54	23.4444
Zm.3883.1.S1_at	2.5706	0.6111	0.3890	9	44.2222
Zm.3895.2.A1_at	1.9588	0.3620	0.5105	52	24.0962
Zm.3906.1.A1_at	2.7294	1.0000	0.3664	5	54.2000
Zm.4089.1.S1_at	2.4706	0.6286	0.4048	15	41.0000
Zm.409.1.S1_at	2.6412	1.0000	0.3786	2	43.5000
Zm.4838.1.A1_at	2.3235	0.5238	0.4304	15	33.8000
Zm.4877.1.S1_at	2.6824	1.0000	0.3728	12	46.6667
Zm.5828.1.A1_at	1.8647	0.3111	0.5363	55	22.8182
Zm.627.1.A1_at	2.5529	0.7628	0.3917	23	32.0000
Zm.6763.1.S1_at	1.9882	0.3963	0.5030	48	25.0833
Zm.704.1.S1_at	2.5471	0.9848	0.3926	12	48.5833
Zm.7091.1.A1_at	2.0706	0.4313	0.4830	45	25.6444
Zm.8365.1.A1_at	2.3412	0.5000	0.4271	21	38.2381
Zm.8773.1.A1_at	2.3059	0.6159	0.4337	24	26.5000
Zm.8927.1.S1_at	0.0000	0.0000	0.0000	0	0.0000
Zm.8972.1.S1_at	2.3471	0.7474	0.4261	20	38.1500
Zm.9463.1.A1_at	2.8235	0.5947	0.3542	20	21.0000
Zm.9645.1.S1_at	2.8471	0.0000	0.3512	1	59.0000
ZmAffx.777.1.A1_at	2.5059	0.9810	0.3991	15	44.4667
Zm.10383.4.S1_a_at	2.2647	0.4507	0.4416	29	31.2759
Zm.15061.1.S1_at	3.8176	0.0000	0.2619	1	20.0000
Zm.16505.1.A1_at	2.7176	0.4803	0.3680	29	20.8621
Zm.4251.1.S1_at	2.9529	0.6952	0.3386	15	21.4000
Zm.4558.1.S1_at	2.9471	0.6618	0.3393	17	20.6471
Zm.5048.6.A1_a_at	2.9529	0.6857	0.3386	15	22.2000
Zm.7545.1.A1_at	3.0294	0.6667	0.3301	4	20.2500
